# Structure and immunological activity of QS-21 variant from *Quillaja saponaria* aerial biomass

**DOI:** 10.3389/fimmu.2026.1771912

**Published:** 2026-03-09

**Authors:** Ricardo San Martín, Yuzhong Liu, Yihan Yang, Christopher B. Fox, L. Ravi Iyer, Raodoh Mohamath, Gabi Ramer-Denisoff, Robert Kinsey, Jeffrey A. Guderian, Jessica Schwabach, Siwen Deng, Natalia de Andrade Teixeira Fernandes, Clayton Radke

**Affiliations:** 1 Sutardja Center for Entrepreneurship and Technology, College of Engineering, University of California, Berkeley, Berkeley, CA, United States; 2 Department of Chemistry, The Scripps Research Institute, La Jolla, CA, United States; 3 Access to Advanced Health Institute (AAHI), Seattle, WA, United States; 4 Department of Biological Engineering, College of Engineering, Utah State University, Logan, UT, United States; 5 Department of Chemical and Biomolecular Engineering, College of Chemistry, University of California, Berkeley, Berkeley, CA, United States

**Keywords:** adaptive immunity, CD4 T cell activation, QS-21, saponin adjuvant, structure-activity relationship (SAR), vaccine adjuvant

## Abstract

QS-21 is a triterpenoid saponin adjuvant component widely used in human vaccines, but its commercial supply is limited because it is sourced almost exclusively from the bark of mature *Quillaja saponaria* trees in Chile. In this study, we report the identification, isolation, as well as structural and immunological characterization of QS-21-Rhamnose (QS-21-Rha), a naturally occurring structural variant that predominates in the leaves and twigs of young *Q. saponaria* shrubs cultivated in California. Analytical profiling showed that QS-21-Rha represents more than 95% of QS-21 variants in the aerial biomass. High-resolution MS/MS and NMR spectroscopy confirmed that QS-21-Rha differs from QS-21 by the C3 terminal rhamnose substitution of xylose. Both *in vitro* and *in vivo* studies demonstrated that QS-21-Rha elicits strong adaptive immune responses, notably robust CD4^+^ T cell activation with immunostimulatory potency comparable to, and exceeding, that of traditionally bark-derived QS-21. From a production standpoint, aerial tissues provide a renewable and higher-yielding source where 1 kg of QS-21 can be obtained from pruning 200 young shrubs, compared with ~1,700 kg of bark, which is equivalent to debarking 100–120 mature trees. These results establish QS-21-Rha as a chemically defined, immunologically active, and more sustainable vaccine adjuvant candidate to potentially address both supply-chain resilience and global vaccine access.

## Introduction

QS-21 is a highly potent, immunostimulatory triterpenoid saponin derived from *Quillaja saponaria* Molina, a tree species endemic to Chile (1, 2). It is a critical component of two of the seven adjuvant formulations currently employed in human vaccines approved by the U.S. Food and Drug Administration (FDA). In particular AS01 is a liposomal adjuvant formulation containing QS-21 that is used in multiple vaccines manufactured by GlaxoSmithKline (GSK), including Shingrix^®^ (herpes zoster) and Arexvy^®^ (respiratory syncytial virus). Moreover, both malaria vaccines currently approved for human use (Mosquirix^®^ and R21/Matrix-M™) as well as the world’s most advanced modern tuberculosis vaccine candidate in Phase 3 clinical development (M72/AS01E) rely on QS-21 as a key adjuvant component. Finally, Novavax’s COVID-19 vaccine (Nuvaxovid) that includes the QS-21 containing Matrix-M™ formulation recently received final FDA approval. Thus, QS-21’s capacity to stimulate both humoral and cellular immunity has resulted in its rapidly expanding use in multiple vaccines for important global health indications (3, 4).

Presently, all QS-21 used in commercial vaccines is sourced exclusively from the bark of mature, wild *Q. saponaria* trees native to Chile. These trees require 25–30 years to develop bark suitable for extraction (2). The traditional harvesting process often destroys the tree, and overharvesting has led to ecological damage and governmental regulations restricting access to these forests, creating an increasingly fragile and unsustainable supply chain.

**Figure 1:**
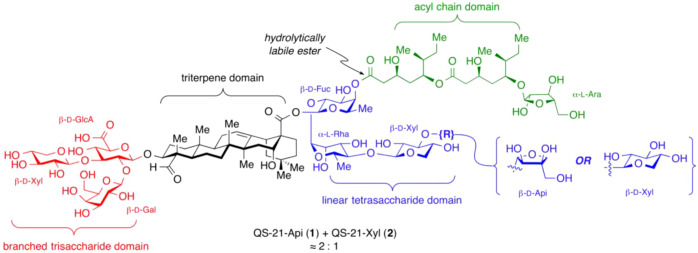
Structure of QS-21 and its four structural domains- adapted from Walkowicz el at. (26).

Structurally, QS-21 derived from bark extracts exists as a heterogeneous mixture of closely related isomers, primarily the canonical QS-21-Xylose and QS-21-Apiose (exact mass of 1988.9242), despite multi-step purification to reach pharmaceutical grade. As shown in **Figure 1**, QS-21 is an amphiphilic triterpenoid saponin, with a molecular structure comprising four structurally distinct regions:

- A branched trisaccharide moiety at the C3 position, typically consisting of D-glucuronic acid, D-galactose, and D-xylose. However, QS-21 variants also exist with L-rhamnose in place of D-Xyl at C-3 (5).
- A quillaic acid triterpenoid core,
- A bridging linear tetrasaccharide at the C28 position, comprised of D-fucose, L-rhamnose, and D-xylose, terminated by either D-apiose or D-xylose, giving rise to two major compositional isomers: QS-21-apiose (QS-21-Api: ~65%) and QS-21-xylose (QS-21 Xyl: ~35%) (6). These saponins are referred to as the canonical QS-21 variants with m/z ~1987.9.
- A hydrolytically labile acyl chain, typically terminated with L-arabinose, is ester-linked to the fucose residue. This ester bond undergoes intramolecular transesterification in basic solution, interchanging between the 3- and 4-hydroxyl groups on the fucose ring, thereby generating two regioisomers, QS-21A and QS-21B, typically in a 20:1 ratio (6).

### Purification of QS-21 from bark extracts

Commercial production of QS-21 currently begins with aqueous extraction of bark harvested from wild *Q. saponaria* trees over 30 years old (2). On a dry weight basis, crude bark extracts contain approximately 5 % w/w total saponins. Following removal of non-saponin components by ultrafiltration, the typical recovery rate is about 60 %, corresponding to a yield ~3 % w/w purified saponins relative to the starting bark material (e.g., Vet-Sap, Desert King International, San Diego, California; ~90 % w/w total saponin content by UPLC). Within this purified mixture, the proportion of QS-21 isomers is variable, averaging 3–5 % w/w as determined by UPLC analysis (2). Further multi-step chromatographic purification yields only ~2 % w/w QS-21 based on the purified saponin fraction (6), which translates to an overall recovery of approximately 0.06 % w/w QS-21 relative to the starting bark. This inherently low yield underscores the severe supply constraints associated with bark-based QS-21 production.

Currently GSK manufactures QS-21 internally under a license with Agenus from semi-purified bark extracts (7). While being a single trisaccharide compositional isomer at the quillaic acid C-3 position (Xyl, as shown in **Figure 1**), the final QS-21 adjuvant product is a mixture dominated by the two C-28 compositional isomers (QS-21-Api and QS-21-Xyl, 65:35 ratio), in addition to structural analogs and process-related impurities. For example, “component 2018” (m/z 2017.9) is a persistent structural impurity that cannot be entirely removed and is treated as an integral part of the product (7). Minor QS-21 variants such as QS-21-Rhamnose (QS-21-Rha, m/z 2001.9) comprise 5–10% of the final formulation. Thus, the regulatory definition of QS-21 is inherently that of a mixed, isomeric product. While recent improvements, such as integrated polar reversed-phase columns and hydrophilic interaction chromatography (HILIC), have increased purity and permitted partial separation of component 2017.9 m/z (6), these procedures are inherently complex, expensive, and do not resolve the intrinsic limitations of supply and isomeric heterogeneity.

At the commercial level, Desert King International (USA), with purification by INDENA (Italy), processes bark from Chile to produce pharmaceutical-grade QS-21 containing primarily canonical forms. Current market prices exceed $100,000-150,000 per gram, reflecting both supply constraints and resource-intensive purification requirements (8).

### Alternative production methods for QS-21

To overcome these bottlenecks, numerous academic and industrial groups have pursued alternative biotechnological solutions, yet all focus on reproducing the canonical QS-21 isomers, without resolving the issues of yield, cost, or scalability. In 2024, Professor Anne Osbourn’s group (John Innes Centre, UK) elucidated and reconstituted the full QS-21 biosynthetic pathway in *Nicotiana benthamiana*, marking the first successful heterologous plant production, though with very low yields that require further optimization for practical application (5, 9, 10). Simultaneously, Professor Jay Keasling’s group (UC Berkeley) achieved *de novo* QS-21 biosynthesis in engineered *Saccharomyces cerevisiae* by introducing 38 genes from six organisms, producing the canonical isomers at titers between 0.1–1 mg/l (10). Thus, even large bioreactors would yield just a few grams per batch, well below commercial needs.

Other approaches, such as plant cell culture methods (e.g., SaponiQx, Lexington, MA), yield similarly low titers (~0.9 mg/l) (11), and result in products functionally equivalent to bark-derived QS-21. Q-Vant Biosciences (Los Angeles, Chile) has developed variable extraction and purification processes from multiple tissues (12), but these are still designed around the apiose/xylose ratio of the canonical forms rather than exploiting other natural variants. Despite improvements in purity and composition, these new platforms still have to address the fundamental issues of low yields, high cost, and supply limitations; as a result, production costs remain comparable to traditional bark-derived QS-21 (13).

### Production and immunological properties of QS-21-rhamnose

Unlike previous work that have concentrated on maximizing yields of the canonical QS-21 isomers, the present work focuses on QS-21-Rhamnose (QS-21-Rha), a structurally distinct, naturally occurring variant defined by the presence of an L-rhamnose residue in the C3-linked trisaccharide in place of xylose.

US 11,254,699 B2 and US 2024/0196770 P1 explicitly document breeding and clonal selection of *Q. saponaria* lines with reduced levels of so-called R-series saponins (those containing C3-rhamnose, including QS-21-Rha), in some cases below 50% of total QS-21 content (14, 15). The stated objective is to simplify downstream purification of X-series (xylose-containing) isomers, thereby aligning raw material composition with existing regulatory approvals and commercial product specifications that are based on bark-derived QS-21. This selective reduction of QS-21-Rha in breeding programs reflects manufacturing and regulatory considerations, rather than an evidence-based assessment of its immunological potential. To date, no studies have reported the isolation of QS-21-Rha in pure form or its evaluation as a vaccine adjuvant.

The present work fills this gap by isolating QS-21-Rha from the leaves and twigs of 2–3-year-old *Q. saponaria* plants cultivated in California and assessing its immunological activity as an adjuvant. A major advantage of this approach is that aerial tissues can be sustainably and repeatedly harvested without harming or destroying the plants, fundamentally shifting QS-21 production from an extractive, low-yield process dependent on the bark of slow-growing, ecologically vulnerable wild trees in Chile to a renewable, high-yield, and cost-efficient platform for generating chemically defined QS-21-Rha. This strategy not only reduces pressure on endangered native populations but also opens new avenues for the scalable and sustainable manufacture of saponin-based vaccine adjuvants.

## Materials and methods

### Saponins in aerial biomass

*Q. saponaria* plants were propagated from seeds collected in October/November 2022 from mature trees found in Berkeley, California. According to historical records from the UC Berkeley Botanical Garden, these trees were introduced to California in the 1930s. Seedlings were cultivated under controlled conditions in the Oxford Tract Greenhouse Facilities at the University of California, Berkeley (Rauscher College of Natural Resources). After one year of growth, a subset of plants was moved to a shaded lath house in 1-gallon pots, while others were planted in the Oxford Tract field. All plants were harvested for analysis at 2.5 years of age. Leaves and twigs were pruned systematically from the upper, middle, and lower canopy of each plant. Fresh biomass (10 g) was thoroughly milled in a coffee grinder and extracted with deionized water at a 5:1 water-to-biomass ratio (v/w). Extractions were performed at 60 °C for 2 hours with manual agitation every 15 minutes. After extraction, the solution was cooled to approximately 10 °C in an ice bath and filtered through Whatman No. 2 filter paper to remove residual plant material. For comparison, leaves from mature trees (greater than 50 years old) found in the UC Berkeley Campus and Botanical Garden were also collected and processed using the same protocol. Additionally, five different lots of dry crushed bark from >30-year-old *Q. saponaria* trees sourced from Chile were extracted under similar conditions, except at a 10:1 water-to-bark ratio (v/w). All extracts were final-filtered through 0.2 μm membranes before quantitative analysis by ultra-performance liquid chromatography–mass spectrometry (UPLC-MS) to determine total saponin and QS-21-Rha content.

### Saponin yield calculations

The saponin content for the samples was estimated using the saponin concentration determined via UPLC, applying a 5:1 volume-to-mass ratio according to the following formula:


% Saponin (w/w) = (Volume (mL) × Concentration (mg/mL)/Sample mass (mg)) × 100

Where:

- Volume = 50 mL (extraction volume)
- Concentration = UPLC-determined saponin concentration (mg/mL)
- Sample mass = 10,000 mg (10 g sample)

For example, for a measured concentration of 20 mg saponin/mL:


% Saponin (w/w) = (50 mL × 20 mg saponin/mL/1000 mg) × 100=10% w/w

This calculation assumes complete extraction efficiency and accounts for the dilution factor inherent in the 5:1 volume-to-mass extraction ratio. However, to estimate realistic yields, the following correction factors must be applied:

1. Extraction recovery: 80% of extract volume recovered.
2. Processing losses: 20% saponin losses during purification.

Therefore, the final estimated yield post-purification in the above example is 6.4% w/w, accounting for both extraction recovery and processing losses.

#### Saponin quantification and identification via UPL/MS

Saponins were analyzed in a UPLC/MS system using a 1260 Infinity II Series System (Agilent, Waldbronn, Germany). The system was equipped with a 100 × 4.6 mm i.d., 2.7 μm particle size, InfinityLab Poroshell 120 EC-C18 column, along with a 4.6 × 5.0 mm i.d. 2.7 μm particle size guard column of the same material (Agilent, Waldbronn, Germany). The column operated at 40 °C. The mobile phase consisted of 0.1% (v/v) formic acid in water (solvent A) and 0.1% (v/v) formic acid in acetonitrile (solvent B). The gradient program was as follows: 30-40% B (3 min), 40–45% B (9 min), 45-100% B (2 min), 100% B isocratic (1 min), and 100-30% B (0.1 min). The total run time was 15.1 min at a flow rate of 1.5 mL/min, with an injection volume of 20 μL. Saponins were monitored at 214 nm. The single quadrupole mass spectrometer was operated in negative ionization mode, scanning a mass range between 750 and 2250 m/z. Nitrogen served as the drying gas at a flow rate of 12.0 L/min and as a nebulizing gas at a pressure of 55.0 psi. The drying gas temperature was set at 350 °C, and the capillary voltage was maintained at 3000 positive and 4500 negative. A calibration curve to quantify total saponins was prepared using the purified product Vet-Sap derived from bark (Desert King International, San Diego, California), that has a saponin content of 90% w/w. Individual saponins were identified based on their m/z ratio using previous reports.^16,21^ Specifically for QS-21-Rha, a thorough analysis was performed in collaboration with the Scripps Lab (see details below).

### Structural analysis of QS-21-Rha

#### Preparative HPLC purification to yield pure QS-21-Rha

From purified spray dried leaf extract (85% saponin content), the QS-21-Rha content determined by UPLC/MS of 11.2%. Purification of the putative QS-21-Rha was performed on a preparative HPLC system (Agilent 1290 Infinity II Prep LC, comprising a G7161B 1290 Prep Binary Pump, G7157A Prep Autosampler, G1365D MWD, and G1364E 1260 FC-PS, Agilent) using the following parameters: solvent A, H_2_O with 0.1% formic acid; solvent B, acetonitrile with 0.1% formic acid. Injection volume: 0.5 mL. Gradient: 60% B from 0 to 2.88 min, 60% to 48.64% B from 2.88 to 8.88 min, 48.64% to 100% B from 8.88 to 9.00 min, hold at 100% B from 9.00 to 14.00 min, return to 40% B from 14.00 to 14.10 min, and hold at 40% B until 19.10 min. The method was performed at a flow rate of 20 mL min^−1^ on an Agilent InfinityLab Poroshell 120 SB-C18 column (21.2 × 150 mm, 4 µm particle size, Agilent). Detection was carried out at 210 nm. Data acquisition and instrument control were performed using Agilent OpenLab CDS software (version 3.7).

#### Liquid chromatography–mass spectrometry

Detection of the putative QS-21-Rha was performed by LC–MS (Agilent 6545 for time-of-flight (TOF), Agilent) using the following parameters: MS (ESI ionization, desolvation line temperature = 250 °C, nebulizing gas flow = 15 l min−1, heat block temperature = 400 °C, spray voltage positive 4.5 kV, negative −3.5 kV). The LC system contained the following modules: G7104A quaternary pump, G7116B thermostatted column compartment, and G7167B autosampler unit (Agilent). Method: solvent A: (H2O + 0.1% formic acid); solvent B: (acetonitrile (CH3CN) + 0.1% formic acid). Injection volume: 10 µl. Gradient: 15% B from 0 to 0.75 min, 15% to 60% B from 0.75 to 13 min, 60% to 100% B from 13 to 13.25 min, 100% to 15% B from 13.25 to 14.5 min, 15% B from 14.5 to 16.5 min. The method was performed using a flow rate of 0.6 ml min−1 and a Kinetex column 2.6 μm XB-C18 100 Å, 50 × 2.1 mm (Phenomenex). Full mass spectra were generated for metabolite identification by scanning within the m/z range of 400–2,500 in negative-ion mode. Analysis was performed using MassHunter Qualitative Analysis v.B.06.00 (Agilent).

#### Tandem mass spectrometry

Purified QS−21−Rha (50 mg L-1) was analyzed using a LC–MS (Agilent 6545 for quadrupole time-of-flight (QTOF), Agilent) in negative ionization mode. The LC system contained the following modules: G1322A solvent degasser, G7120A binary pump, G1316C thermostatted column compartment, and G7167B autosampler unit (Agilent). Metabolites were separated using an Agilent 1290 series HPLC installed with a Kinetex 2.6 µm XB-C18 100 Å Column 50 x 2.1 mm column (00B-4496-AN, Phenomenex, Torrance, CA, USA. Mass spectra were generated using the same LC–MS parameters with a collision energy of 120 V. Analysis was performed using MassHunter Qualitative Analysis v.B.06.00 (Agilent).

#### NMR spectroscopy

Purified QS−21−Rha (10 mg) was characterized by 1D and 2D NMR (¹H, ¹³C, HSQC) in acetonitrile-d3:D2O 1:1 at 298 K on a Bruker 600 MHz NMR spectrometer equipped with a cryoprobe. Sugar connectivity and terminal methyl identification leveraged anomeric chemical shifts, coupling constants, HSQC, HMBC, and HSQS-TOCSY correlations.

### Immunological tests of QS-21-Rha

All immunological assays were conducted at the Access to Advanced Health Institute (AAHI, Seattle, Washington) under the guidance of Dr. Christopher Fox, using purified QS-21-Rha supplied by the UC Berkeley team. For all comparative studies, canonical QS-21 purified by AAHI from commercial bark extracts served as the reference standard (6). Throughout this manuscript, QS-21-Rha purified by UC Berkeley is also referred to as “QS-21-Rha,” whereas the canonical bark-derived QS-21 is denoted as “QS-21.”

#### Source of QS-21-Rha

The biomass was air dried and milled to a particle size of 1 mm and extracted with deionized water (10:1 w/w ratio of water to ground leaves) at 60 °C for 2 hours under constant agitation. Following extraction, the suspension was cooled, and solid debris removed by sequential passage through a 75 μm sieve and filtration with Whatman No. 2 filter paper. The clarified extract was then diafiltered using 20 kDa molecular weight cutoff membranes, employing a 5:1 v/v ratio of deionized water to extract, to reduce low molecular weight impurities. The resulting retentate was freeze-dried to yield a purified saponin-rich powder. UPLC/MS analysis of the powder indicated a total saponin content of 85% w/w, QS-21-Rha accounting for approximately 10% w/w of the total saponin fraction.

#### Purification of QS-21-Rha

to obtain the final purified product, a UPLC/MS system using a 1260 Infinity II Series System (Agilent, Waldbronn, Germany) was used. The system was equipped with a Zorbax Poroshell 120 EC-C18 Semi Preparative 9.4 mm x 100mm 2.7-Micron. The column operated at 40 °C. The mobile phase consisted of 0.1% (v/v) formic acid in water (solvent A) and 0.1% (v/v) formic acid in acetonitrile (solvent B). The gradient program was as follows: 30-40% B (3 min), 40–45% B (9 min), 45-100% B (2 min), 100% B isocratic (1 min), and 100-30% B (0.1 min). The total run time was 15.1 min at a flow rate of 1.5 mL/min, with an injection volume of 20 μl. Saponins were monitored at 214 nm. The single quadrupole mass spectrometer was operated in negative ionization mode, scanning a mass range between 750 and 2250 m/z. Nitrogen served as the drying gas at a flow rate of 12.0 L/min and as a nebulizing gas at a pressure of 55.0 psi. The drying gas temperature was set at 350 °C, and the capillary voltage was maintained at 3000 positive and 4500 negative.

To enhance process efficiency, two UPLC/MS methods were developed. The first method, applied during the initial enrichment step, employed a longer gradient profile to separate QS-21-Rha from other co-extracted components due to the relatively high impurity load. The second method, optimized for use in subsequent processing stages, utilized a shortened gradient suitable for enriched samples.

**Figure 2:**
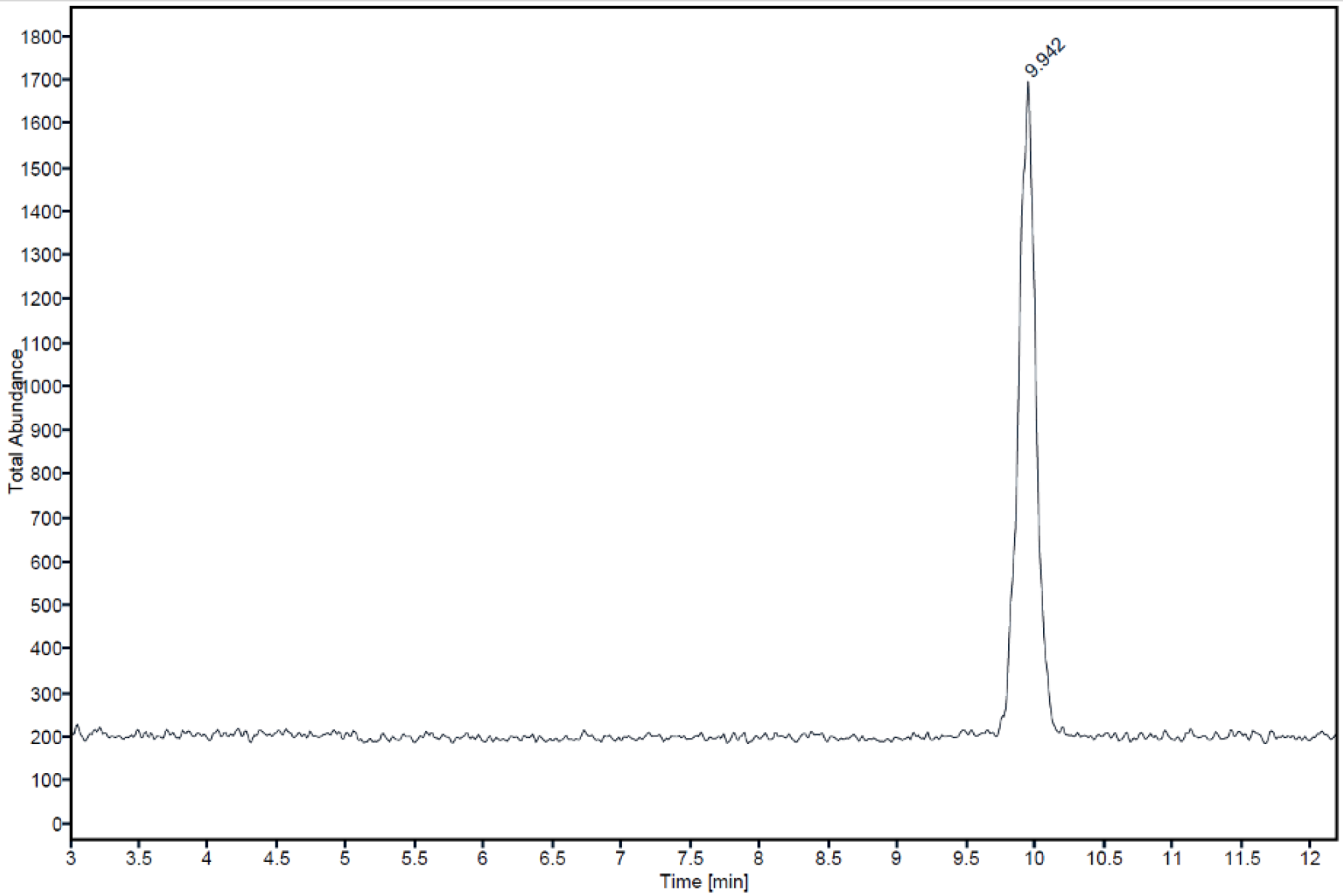
MS signal for the final purified QS-21-Rha saponin.

A series of preparative fractionation cycles was performed using injections of 800 µL per run of the concentrate, with 5 mL of eluate collected per injection. The combined eluates were pooled and analyzed via UPLC/MS, yielding a QS-21-R purity of 75% w/w of total saponins. This intermediate mixture was subjected to a second round of purification using a more selective chromatographic method. In this step, 800 µl injections were employed, and 2.5 ml of eluate was collected per injection. The collected fractions were again combined and analyzed by UPLC/MS, confirming a final QS-21-Rha purity >98%. **Figure 2** shows the MS signal for the final product with a purity > 98%. The final pooled fractions were lyophilized to remove water and concentrate the purified QS-21-R, rendering the material suitable for subsequent *in vitro* and *in vivo* immunological assays.

#### Vaccine raw materials

QS-21-Bark was purified by Infectious Disease Research Institute (now Access to Advance Health Institute, Seattle, WA) as described (6). 1,2-dioleoyl-sn-glycero-3-phosphocholine (DOPC) was purchased from Lipoid LLC (Ludwigshafen, Germany). Cholesterol was purchased from Wilshire Technologies (now Evonik Industries AG, Germany). Ammonium phosphate monobasic and ammonium phosphate dibasic were purchased from J. T. Baker (now Avantor, Allentown, PA). GLA was purchased from Avanti Polar Lipids (Alabaster, AL), and ID93 was obtained from Biovac (Cape Town, South Africa).

#### Formulation of liposomes

QS-21-Bark or QS-21-Rha was formulated into liposomes for immunological evaluation using established lipid film hydration and microfluidization protocols. Specifically, a lipid mixture containing 0.04 mg/mL QS-21-Bark or QS-21-Rha, 8 mg/mL DOPC, and 2 mg/mL cholesterol was prepared in 25 mM ammonium phosphate buffer at pH 5.7, with a total batch volume of 50 mL. The powdered components were first dissolved in chloroform, then the solvent was evaporated under reduced pressure (≤25 mbar) using a rotary evaporator to form a uniform dry lipid film. This film was subsequently rehydrated with buffer by sonication at 60 °C for approximately 1 hour to facilitate liposome formation. The resulting suspension was then homogenized using an LM20 high-pressure microfluidizer (Microfluidics Corp., Westwood, MA) at 20,000 psi for five consecutive passes to ensure uniform particle size. Finally, the liposome preparation was filtered through a 0.2 μm membrane to remove aggregates and ensure sterility, yielding a monodisperse population of QS-21-containing liposomes suitable for immunological assays. Blank liposomes that did not contain QS-21 were also prepared as a negative control. QS-21-containing or blank liposomes were admixed with GLA-AF and recombinant tuberculosis antigen ID93 immediately before immunizations (**Supplementary Table 4**). GLA-AF was prepared as described previously (16).

#### Particle size assay

The average particle diameter of the formulated liposomes was analyzed by dynamic light scattering (DLS) using a Zetasizer Nano ZS (Malvern Instruments, Worcestershire, UK). Liposome samples were diluted 1:100 in Milli-Q water and were measured three to nine times at 25 °C and a scattering angle of 173°. Data acquisition and analysis were performed using Malvern DTS v.7 or later software.

#### *In vitro* human whole blood cytokine assay

QS-21-containing liposomes prepared as described above were diluted to saponin concentrations of 8, 4, 2, and 1 μg/mL and evaluated in a human whole blood cytokine assay. Fresh heparinized whole blood from six healthy adult donors (3 males and 3 females) was obtained from Bloodworks Northwest (Seattle, WA). Optimal cytokine induction was observed at 4 μg/mL QS-21. For analysis, 50 mL of peripheral blood was collected into sodium heparin tubes (10 mL each) on the day of the assay. Liposome formulations—including control canonical bark-derived QS-21 liposomes and leaf-derived QS-21-Rha liposomes, were dispensed at 4 μg/mL (50 μL/well) into 96-well plates. Negative controls consisted of blank liposomes (no QS-21) and saline. Whole blood (200 μL) was added to each well, and the plates were incubated at 37°C for 24 hours. Following incubation, plasma supernatants were collected and analyzed for monocyte chemoattractant protein-1 (MCP-1) cytokine levels using standard immunoassay methods (Invitrogen #88-7399-88).

#### *In vivo* immunogenicity assays

All animal studies were approved by and performed in accordance with the Bloodworks Northwest Research Institute’s Institutional Animal Care and Use Committee (Seattle, WA), protocol #5389-02. C57BL/6 mice (The Jackson Laboratory, Harbor, ME), 6–8 weeks old, divided evenly by sex, were used for experiments (*n* = 4/group for the saline control and *n* = 8/group for other experimental groups). The mice were anaesthetized and bilaterally immunized intramuscularly at days 0 and 21 according to **Supplementary Tables 4, 5**. Blood samples for serum analysis of antibody titers were taken on days 0 and 21 by retro-orbital bleed. An experimental error occurred on day 35 resulting in approximately half of the study mice being injected with an unrelated formulation. The error was immediately identified, and all of the mice were sacrificed on day 35 to avoid any immunological impact of the experimental error. On the day 35 endpoint, mice were euthanized via CO_2_ and cervical dislocation, followed by a terminal bleed and harvesting of spleens and bone marrow for immunological analysis. Serum and tissues were harvested and stored on ice immediately after euthanasia. Fractionated serum samples were then stored at -80 °C until ELISA analysis. Cell and tissue samples were processed on the same day as harvest.

#### Flow cytometry

Harvested spleens were processed into single-cell suspensions by manual disruption, pressing the organ through a 70-µm filter screen using a syringe plunger, followed by red blood cell lysis and washing. The cells were counted and plated into 96-well round-bottom plates, followed by stimulations. Negative controls were incubated in media alone, positive controls were incubated in the presence of PMA-ionomycin (eBioscience Cell Stimulation Cocktail 500X, Invitrogen #00-4970-93), and experimental wells were incubated with recombinant ID93 antigen (Biovac Lot# 17ID93002p) at a final concentration of 10 µg/mL. The PMA-ionomycin stimulations were performed on splenocytes pooled in equal numbers from all mice within each experimental group. Two hours into the stimulation, brefeldin A (BD GolgiPlug #555029) was added to the wells, and the cells were stimulated for an additional 6 hours before being held at 4 °C overnight prior to staining for flow cytometry. Cell viability and surface staining was performed first using the following antibodies and stains at the following dilutions: LIVE/DEAD Fixable Blue Dead Cell Stain for UV excitation (Thermo #L23105, 1:500), anti-CD16/32 (“Mouse FcBlock”, BD #553141, Clone 2.4G2, 1:100), anti-CD4-BV605 (BD #563151, Clone RM4-5, 1:200), anti-CD3-PerCP-Cy5.5 (Invitrogen #45-0031-82, Clone 145-2C11, 1:100), anti-CD8-ef450 (Invitrogen #48-0081-82, Clone 53-6.7, 1:200), anti-CD44-AF488 (BioLegend #103016, Clone IM7, 1:200), and anti-CD154-BV750 (BD #747495, Clone MR1, 1:100). After surface staining, the samples were fixed for 15 minutes in BD Cytofix/CytoPerm (BD #554714) and stained for intracellular cytokines. The following antibodies were used at the following dilutions: anti-TNFa-BV480 (Invitrogen #414-7321-82, Clone MP6-XT22, 1:100), anti-IL-2-APC (Invitrogen #17-7021-82, Clone JES6-5H4, 1:100), anti-IL-17a (BioLegend #506914, Clone TC11-18H10.1, 1:100), anti-IL-5-PE (BD #554395, Clone TRFK5,1:100), and anti-IFNg-PE-Cy7 (BioLegend #505826, Clone XMG1.2, 1:100). The cells were then washed and resuspended in PBS + 1% BSA + 1 mM EDTA for flow cytometry analysis on a Beckman-Coulter Cytoflex LX flow cytometer. Results were analyzed using FlowJo software (Tree Star Inc, Ashland, OR). The gating strategy with representative samples are shown in **Supplementary Figure 9**. The prevalence of cytokine and activation marker positive cells are expressed as the percentage of CD4^+^CD44^+^ or CD8^+^CD44^+^ cells.

#### ELISAs & ELISpots

Antigen-specific total IgG (IgGT), IgG1, and IgG2c were measured in serum samples from the immunized animals. Briefly, ELISA plates were coated with 1 µg/mL ID93 protein followed by the addition of serially diluted serum and subsequently horseradish peroxidase (HRP)-conjugated detection antibodies against mouse IgG (SouthernBiotech #1031-05), IgG1 (SouthernBiotech #1070-05), or IgG2c (SouthernBiotech #1079-05). ELISA plates were developed using a 3,3’,5,5’tetramethylbenzidine (TMB) substrate (Invitrogen #5120-0083) and stopped with H_2_SO_4_. Endpoint titers were quantified by a least-squares fit of A450 data to a 4-parameter sigmoidal curve, using a cutoff established by serum samples from naïve animals. Titer values that could not be quantified were set at half of the assay’s lower limit of detection.

Antigen-specific antibody-secreting cells in murine bone marrow samples and cytokine-secreting mouse splenocytes were both quantified using ELISpot assays. Briefly, multiscreen ELISpot plates (Millipore #MSIPS4W10) were coated with a capture ligand: either 2 µg/mL of ID93 for bone marrow IgG ELISpots, or anti-mouse IFNγ (BD Biosciences #551881), IL-5 (BD Biosciences #551880), or IL-17a (R&D Systems #EL421) for splenocyte ELISpots. Homogenized bone marrow or splenocyte tissue cell isolates were incubated on the ELISpot plates for 3–72 hours. Plates were developed using HRP-conjugated detection antibodies (eBioscience #18-4100-94) and 3-amino-9-ethylcarbazole (AEC) substrate kits (Vector Laboratories, Newark, CA) according to the manufacturer’s protocol. The reaction was stopped by washing the plates with deionized water before being dried in the dark. Positive spots were enumerated using an automated ELISpot reader (CTL Analyzer, Cellular Technology Limited, Cleveland, OH). Data were analyzed using ImmunoSpot software (Cellular Technology Limited).

### Statistical analyses

Statistical analyses were conducted using GraphPad Prism v10. *In vitro* WBA, ELISA, and ELISpot data were log-transformed and analyzed with ordinary one-way ANOVA with Tukey’s or Sidak’s correction for multiple comparisons. Flow cytometry data were analyzed by ordinary one-way ANOVA with Tukey’s test for multiple comparisons.

## Results and discussion

### Saponin composition in aerial biomass vs bark of mature trees

**Figure 3:**
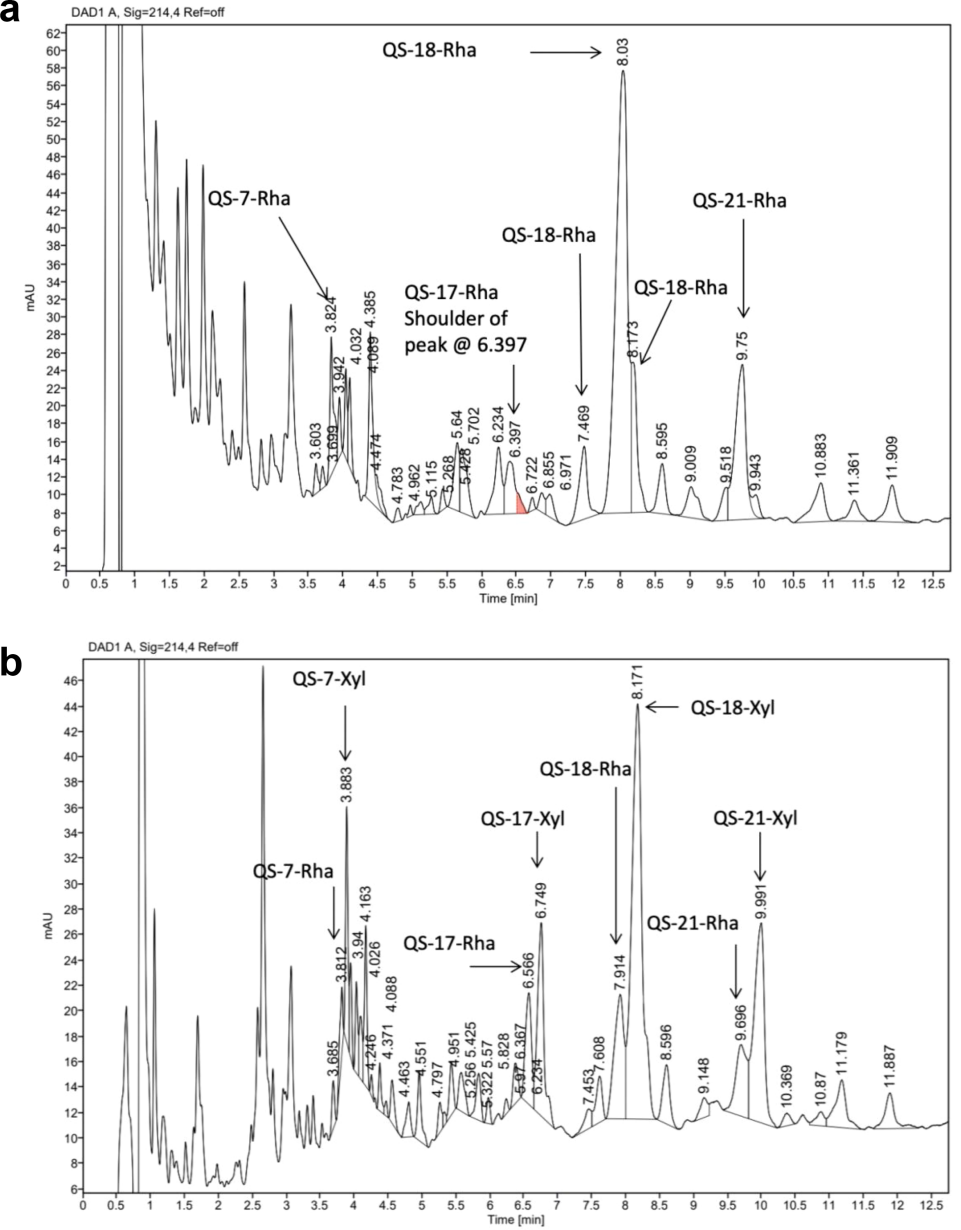
**(A)** UPLC chromatograms of a leaf and twigs extract versus **(B)** a bark derived extract. See text for discussion on the saponins corresponding to each peak.

**Figure 3A** presents a typical UPLC chromatogram of an aqueous extract from leaves and twigs of 2–3-year-old *Q. saponaria* shrubs. UPLC/MS analysis consistently revealed QS-18-Rha as the dominant constituent (peaks min 7.469, 8.03, and 8.173, m/z 2165.8; 2166.2; 2164.8 respectively), followed by the putative QS-21-Rha at min 9.75, m/z 2003.4. The latter species matches compounds S3 and S5 (17), characterized by the presence of a rhamnose residue in the C-3 trisaccharide and either β-D-xylose or β-D-apiose at the C-28 glycosyl position. This compound was confirmed to be QS-21-Rha through extensive analytical work. Importantly, only trace levels of the canonical QS-21 isomers (m/z 1988), which are typically abundant in bark of mature trees (over 30 years old), were detected in leaf extracts. Moreover, QS-7 and QS-17 corresponded to QS-7-Rha and QS-17-Rha, based on their m/z values of 1877.2 and 2310, respectively. In all cases, the mass difference between the xylose and rhamnose version corresponds to 14.

In contrast, **Figure 3B** shows the UPLC chromatogram of a bark extract. Here, the chromatographic profile corresponding to QS-21 exhibits increased complexity, reflecting the presence of co-eluting saponin variants and non-target compounds characteristic of crude extracts. **Supplementary Table 1** summarizes the principal saponins identified through UPLC/MS analysis (**Figure 2**). The data clearly demonstrate that bark extracts from mature trees contain a heterogeneous mixture of both apiose and xylose variants of QS-7, QS-18, and QS-21, along with elevated concentrations of QS-17. This compositional profile is consistent with the original findings of Kensil et al. (1). and aligns with the heterogeneous saponin composition described in GSK’s patent application WO2019/106192A1 (7), which acknowledges the fact that the QS-21 of bark-derived extracts is more complex than that of leaf-derived extracts, difficulting its purification procedure.

### Structural analysis of QS-21-Rha

**Figure 4:**
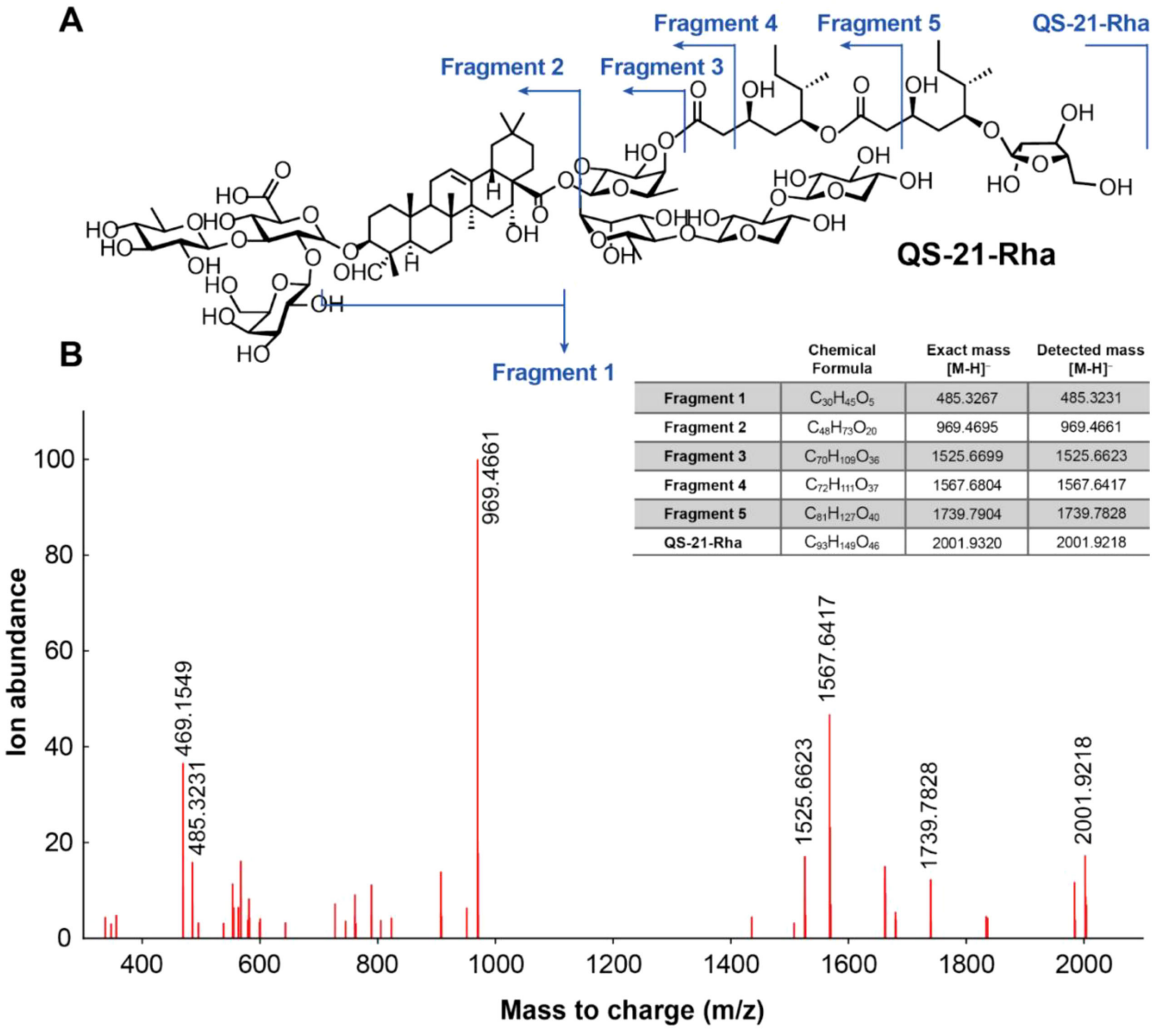
Structural characterization of QS-21-Rha by high-resolution tandem mass spectrometry. **(A)** Structure of QS-21-Rha annotated with key diagnostic MS/MS fragment ions. The molecular ion [M–H]^−^ was observed at m/z 2001.9218. Fragmentation yielded characteristic ions at m/z 969.4661 (trisaccharide fragment), 1525.6363, 1567.6623, 1739.7828, and 485.3231, consistent with sequential glycosidic cleavages and loss of the acylated sugar chain. **(B)** High-resolution negative-ion MS/MS spectrum of QS-21-Rha highlighting the observed monoisotopic peaks. The fragmentation pattern confirms substitution of rhamnose at the C3 glycoside position and matches the expected structural features of QS-21-Rha.

High-resolution ESI-MS analysis of the purified saponin revealed a molecular species at m/z 2001.9218 [M–H]^-^, corresponding to the intact saponin. This value is 14 Da higher than that of QS-21, immediately suggesting substitution of a pentose by a deoxyhexose residue. In the tandem mass spectrum, a diagnostic trisaccharide fragment was detected at m/z 969.4661 for the current compound of interest, whereas in QS-21 the corresponding fragment appears at m/z 955.4539 (**Figure 4**). Because the quillaic acid aglycone is unchanged between QS-21 and QS-21-Rha, the mass shift must arise within the C3 glycosidic substituents. The observed 14.0122 Da difference between these ions matches the expected mass increment for α-L-rhamnose (164.0685 Da) replacing β-D-xylose (150.0528 Da) thereby pinpointing the terminal sugar substitution of rhamnose in QS-21-Rha instead of xylose in QS-21.

Additional 1D and 2D NMR studies (i.e. 1H, 13C, HSQC, HSQC-TOCSY, HMBC) further confirmed this structural assignment (**Supplementary Figures 1-6**; **Supplementary Table 2**). Compared to QS-21, an additional ¹H methyl doublet in the ~1.1–1.3 ppm region and its correlated ¹³C resonance near ~17–18 ppm indicates the presence of an additional methyl group (rhamnose compared to xylose). HSQC-TOCSY traces the full deoxyhexose spin system, confirming the methyl attachment to a sugar moiety. Critically, HMBC displays a three-bond correlation from the rhamnose anomeric H-1 to the GlcA C3, establishing the 1–3 glycosidic linkage. The remainder of the oligosaccharide framework (including the conserved C28 chain) shows connectivity consistent with QS-21 reference data (5). Together, the combination of tandem MS and NMR studies confirm that the structural change is the C3 terminal rhamnose instead of xylose compared to QS-21.

### Abundance of QS-21-Rha in aerial biomass of young *Q. saponaria* plants

**Supplementary Table S3** summarizes the total average saponin content in leaves and twigs of 2.5-year-old *Q. saponaria* shrubs (values unadjusted for extraction or purification losses). Total saponins were 10.1 % for field-grown plants, 7.0 % for lath-house plants, and 11.2 % for greenhouse plants. Within these totals, the average proportion of QS-21-Rha was 23.2 % (field), 20.6 % (lath), and 12.9 % (greenhouse). Expressed on a dry biomass basis, these values correspond to QS-21-Rha yields of 2.3 % for field-grown, 1.4 % for lath-grown, and 1.5 % for greenhouse-grown shrubs. In comparison, leaves from mature trees (>50 years old) collected on the UC Berkeley campus and in Santa Barbara, California contained 2.2–4.0 % total saponins with 14–16 % QS-21-Rha, giving only 0.3–0.6 % QS-21-Rha per dry weight. Bark samples contained an average of 5 % total saponins (w/w), of which approximately 5 % were canonical QS-21 isomers. This corresponds to a yield of only ~0.25 % QS-21 relative to the dry weight of bark, without considering extraction or purification losses.

Altogether, these findings show that QS-21-Rha accumulation in young shoots is approximately 6–9-fold higher than canonical QS-21 content typically found in bark. While QS-21-Rha has previously been reported as a minor component in bark (4, 12, 14) and a major variant in leaf and twig tissues (5), it has not been previously isolated in pure form or evaluated for adjuvant activity, as described in this work.

Notably, the compositional profiles of aerial biomass and bark extracts are essentially reversed. Bark-derived QS-21 preparations contain 65–70 % canonical isomers and only 5–10 % QS-21-Rha (7), whereas leaf- and twig-derived material consists of >98 % QS-21-Rha with only trace amounts of canonical forms. The predominance of QS-21-Rha in young leaves and twigs, compared to the canonical xylose-containing QS-21 in mature bark, is probably linked to differences in glycosyltransferase activity during plant development. Two key UDP-dependent glycosyltransferases UGT73CX1 (xylosyltransferase) and UGT73CX2 (rhamnosyltransferase) were previously identified to attach xylose or rhamnose, respectively, to the C-3 position of the quillaic acid scaffold (5).

In summary, the dominance of QS-21-Rha in aerial tissues and QS-21-Xyl in bark is probably a direct consequence of tissue- and stage-specific glycosyltransferase expression and sugar-nucleotide availability in *Q. saponaria*.

### Immunological testing of QS-21-Rha

After the isolation of high-purity QS-21-Rha from *Quillaja saponaria* leaves and the elucidation of its structure, its immunological properties were evaluated as a vaccine adjuvant when formulated into liposomes. These properties were then compared with those of canonical QS-21 formulated under the same conditions. The following sections describe the results of this evaluation.

### Formulation and *in vitro* immunological test with QS-21-Rha

QS-21-Rha was formulated in conventional DOPC-cholesterol liposomes prepared by high-pressure homogenization. The QS-21-Rha liposome particle diameter (Z-ave) was 93.3 +/- 0.4 nm with a polydispersity index (PdI) of 0.224 +/- 0.008. Comparable physical characteristics were demonstrated by the canonical QS-21 liposomes (75.5 +/- 1.7 nm Z-ave and 0.274 +/- 0.024 PdI) and the blank liposomes (83.3 +/- 1.7 nm Z-ave and 0.239 +/- 0.007 PdI).

Innate immunological response of cells in human whole blood was tested when stimulated *in vitro* with the liposomes containing QS-21-Rha. **Supplementary Figure 7** shows monocyte chemoattractant protein 1 (MCP-1) production as an indicator of innate immune stimulation from liposomes containing 4 µg/mL of either canonical QS-21 (bark-derived, purified by AAHI essentially as described previously (6) or QS-21-Rha (leaf-derived, purified by UC Berkeley). Both formulations elicited a statistically significant increase in cytokine production compared to the saline control. Importantly, the MCP-1 levels induced by QS-21-Rha liposomes were not significantly different from those elicited by canonical QS-21 liposomes.

In conclusion, the *in vitro* immunogenicity assay demonstrated that the purified single-isomer QS-21-Rha appeared comparably immunostimulatory to bark-derived QS-21 in the context of human whole blood, with no statistically significant difference in MCP-1 stimulation activity between the two formulations. Given these findings, and consistent with the well-established safety and adjuvant activity profile of canonical QS-21, we next considered whether the new leaf-derived isomer exhibited similar immunological behavior *in vivo*.

### *In vivo* immunological test with QS-21-Rha

To expand upon the *in vitro* findings, a limited *in vivo* mouse immunogenicity assay was conducted. Human whole blood assays are an established preclinical model for evaluating early innate-immune activation and adjuvant safety in a physiologically relevant environment, and the observed equivalence between QS-21-Rha and canonical QS-21 provided a direct indication that the new isomer would behave similarly *in vivo*. Importantly, canonical QS-21 has been extensively characterized in preclinical and clinical settings, consistently demonstrating potent adjuvant activity, well-defined mechanisms of action, and an established safety profile (18–21). This substantial existing literature provides strong precedent for the expected immunological behavior and tolerability of QS-21-based adjuvants. Taken together, the (i) human whole blood data showing appropriate innate activation without excessive inflammatory signatures and (ii) the extensive published evidence supporting the safety and efficacy of canonical QS-21 provided the minimal ethical rationale required to justify a focused, preliminary *in-vivo* study of the newly identified QS-21-Rha isomer. The purpose of the mouse study was therefore to determine whether the innate functional equivalence observed *in vitro* translated to organism-level immunogenicity and to guide the direction of future mechanistic and dose-response studies.

The *in vivo* mouse immunogenicity assay was performed to directly compare the adjuvant activity of the QS-21-Rha liposomes with that of the canonical QS-21 liposomes when paired with a recombinant vaccine antigen. Moreover, since QS-21 is known to synergize with Toll-like receptor 4 (TLR4) agonists for optimal combination adjuvant activity as in AS01 and similar adjuvant systems (22, 23), we also added synthetic TLR4 agonist glucopyranosyl lipid adjuvant aqueous nanosuspension (GLA-AF) to the liposome preparations for comparison. All adjuvant formulations were paired with the clinical-stage recombinant tuberculosis vaccine antigen ID93 at a 0.5-µg dose. Canonical QS-21 (containing primarily the apiose/xylose isomers) and UC Berkeley-sourced QS-21-Rha were both employed at a dose of 2 μg per mouse; where included, GLA was dosed at 5 μg per mouse. Blank liposomes served as negative controls and were paired with the ID93 antigen but neither QS-21 nor GLA. **Supplementary Table 4** summarizes the vaccine formulations employed for this experiment, enabling a side-by-side assessment of the immunological potency and profile conferred by each adjuvant system.

C57BL/6 mice (4–8 per group) were intramuscularly immunized twice, 21 days apart, with the vaccine preparations described in **Supplementary Tables 4, 5**. Antigen-specific immune responses were assessed two weeks following the second immunization. **Supplementary Table 5** outlines the timeline and readouts used in the experiment.

**Figure 5:**
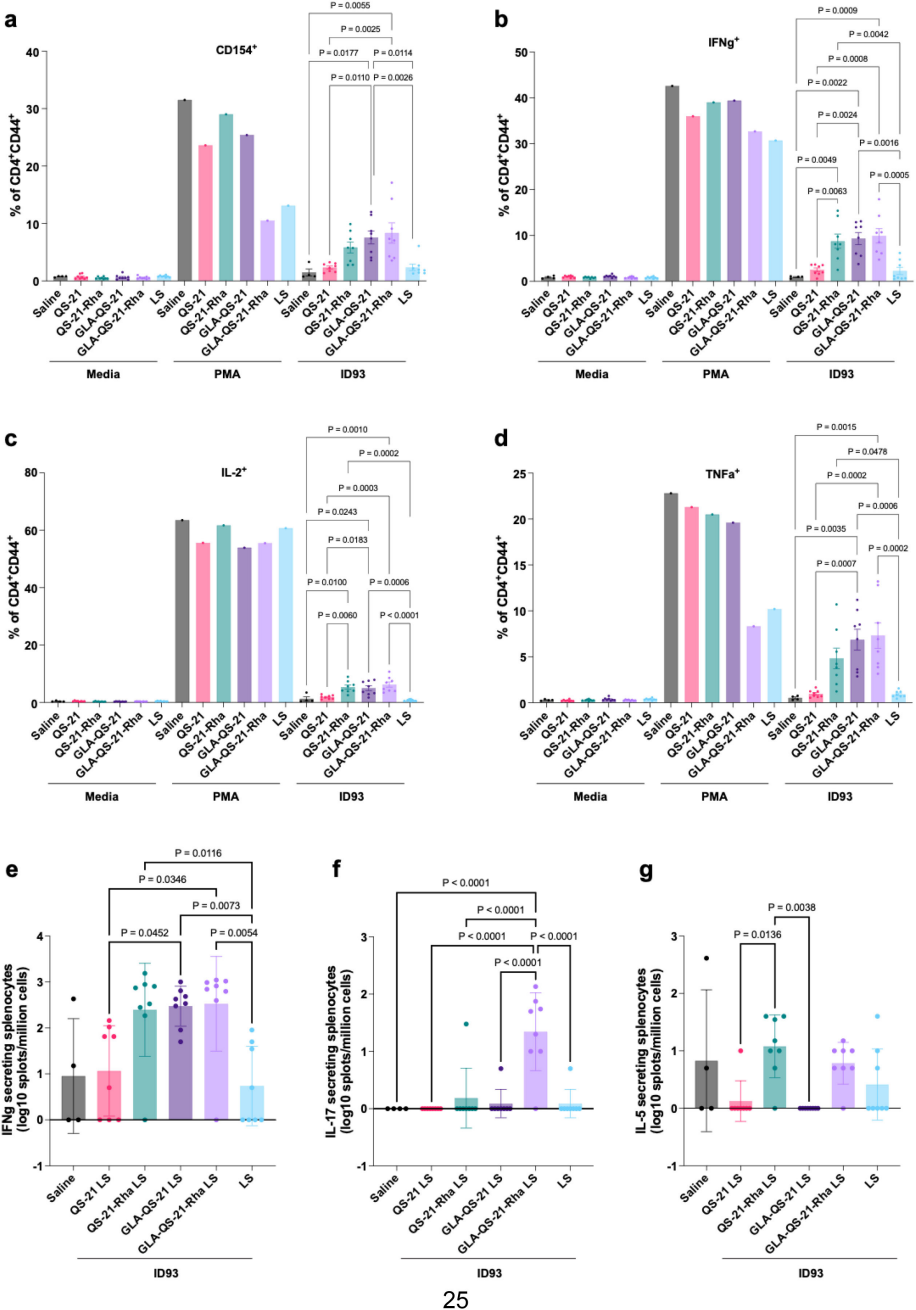
Antigen-specific cellular immune responses in mice immunized with ID93 + QS-21-Rha liposomes (LS) vs QS-21 liposomes (LS). **(A-D)** Adaptive ID93-specific CD4^+^ T cell responses and **(E-G)** cytokine-secreting splenocytes. C57BL/6 mice (*n* = 8/group except for saline control with *n* = 4/group) were immunized on days 0 and 21, and spleens were harvested on day 35. **(A-D)** Medium was used for negative controls, and PMA-ionomycin was used for positive controls. Means and SEM are shown. Statistical analysis by ordinary one-way ANOVA with Tukey’s test for multiple comparisons. **(E-G)** Bars show mean and SD. Data were log-transformed and analyzed by one-way ANOVA with Tukey’s correction for multiple comparisons.

ID93-specific cellular immune responses in spleens were analyzed by intracellular cytokine staining with flow cytometry and ELISpot. CD154 (CD40L) is an important marker of T cell activation notably expressed on follicular helper T_fh_ cells where it provides co-stimulation for B cell activation and the induction of antibody responses. As seen in **Figures 5A**, the QS-21-Rha liposomes trended towards inducing greater but non-significant percentages of CD154+ ID93-reactive CD4+ T cells than the canonical QS-21 liposomes. The addition of the TLR4 agonist GLA to the formulations increased responses marginally in the case of the QS-21-Rha liposomes (where response was already elevated) and significantly in the case of the canonical QS-21 liposomes compared to each liposome formulation without the GLA.

IFNγ (interferon gamma) is a critical antiviral cytokine produced by activated T cells. As shown in **Figures 5B**, the QS-21-Rha liposomes induced significantly greater percentages of IFNγ+ memory CD4+ T cells than the canonical QS-21 formulation. The addition of GLA did not increase this response significantly for the QS-21-Rha liposomes since there was already substantial response without GLA. The canonical QS-21 liposomes induced significantly greater percentages of IFNγ+ CD4+ memory T cells than saline or blank liposome controls only when GLA was included.

IL-2 is a cytokine produced by activated T cells which acts in multiple ways including immune cell proliferation and enhancement of cytotoxic T and NK cell killing to control the adaptive immune response. Results in **Figure 5C** largely mirrored that of the IFNγ response detailed above, with the QS-21-Rha liposomes inducing a greater IL-2+ CD4+ T cell response than the canonical QS-21 liposomes. As seen with IFNγ, addition of GLA to the QS-21-Rha liposomes did not significantly increase the CD4+ IL-2 response to ID93 in contrast to the pattern evident with the canonical QS-21 liposomes.

TNFα is a key multifunctional cytokine produced and secreted by activated T cells. Results in **Figure 5D** were comparable to the results described above for IFNγ and IL-2 with the QS-21-Rha liposomes, but not the canonical QS-21 liposomes, inducing significantly greater TNFα-producing CD4+ cells compared to the blank liposomes. Again, addition of GLA to the formulation significantly increased the response induced by the canonical QS-21 liposome formulation, but only marginally increased the already elevated response of the QS-21-Rha liposomes.

Responses to other cytokines measured, including IL-5 (indicative of a Th2-quality response) and IL-17 (representing a Th17-quality response) were not substantive for any formulation tested (**Supplementary Figure 8**). Likewise, no notable CD8+ T cell responses were detected (data not shown).

As an orthogonal assessment of cellular immune responses, we enumerated ID93-specific cytokine-producing splenocytes by ELISpot. IFNγ-secreting splenocyte results (**Figure 5E**) were consistent with the intracellular cytokine data for CD4+CD44+ cells generated by flow cytometry analysis in **Figure 5B**. However, in contrast to the flow cytometry analysis, there appeared to be some IL-17 and IL-5 responses in some experimental groups when assayed by ELISpot. In particular, the GLA-QS-21-Rha liposomes elevated IL-17 response compared to all other groups, and the QS-21-Rha liposomes showed some IL-5 response compared to the canonical QS-21 liposomes although this was not significantly different from saline or blank liposome controls (**Figures 5F, G**).

Overall, the QS-21-Rha liposomes induced a greater magnitude CD4+ T cell response than the canonical QS-21 liposomes, and this response reflected Th1 polarization with significant proportions of IFNγ-, IL-2- and TNFα-positive cells. Adding the TLR4 agonist to the canonical QS-21 liposomes substantially enhanced responses, consistent with our previous experience (22, 23). The addition of GLA to the QS-21-Rha liposomes also tended toward increasing response magnitude, but did not reach statistical significance since the response was already elevated in the absence of GLA. This contrasts with the pattern evident between the canonical QS-21 liposomes alone compared with when GLA was added, suggesting that the QS-21-Rha liposomes operate in a distinct manner to the canonical QS-21 formulation, with the potential for inducing greater cellular immune responses in the absence of a TLR4 agonist. Finally, the ELISpot assays further suggest that the QS-21 Rha formulation may possess cellular immunostimulatory qualities distinct from the canonical QS-21 liposomes, warranting further studies to confirm and expand on these results.

**Figure 6:**
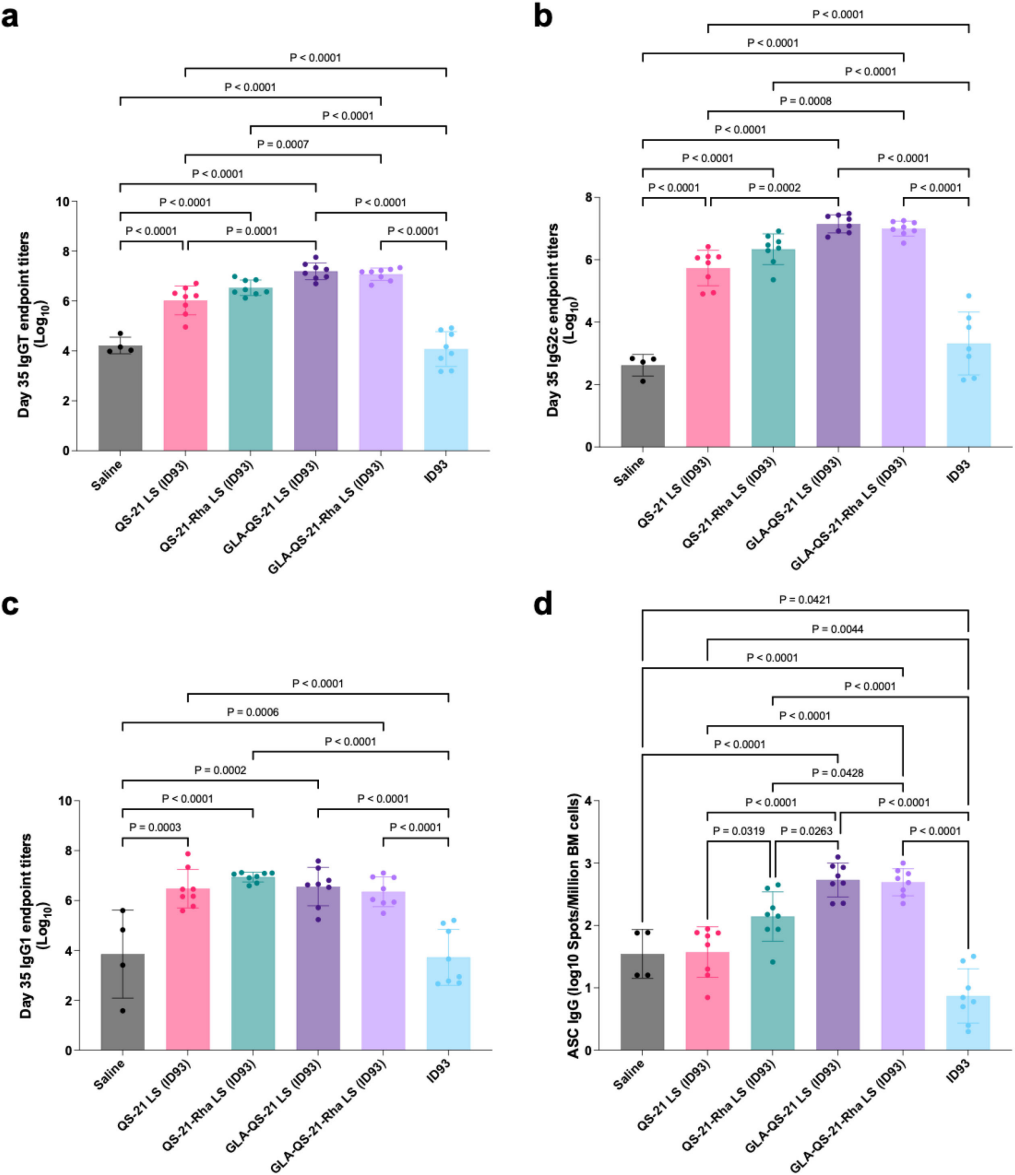
Antigen-specific antibody responses from immunized mice. C57BL/6 mice (*n* = 8/group except for saline control with *n* = 4/group) were immunized on days 0 and 21, and serum and bone marrow were harvested on day 35. **(A-C)** Serum antigen-specific antibody titers: **(A)** Total IgG, **(B)** IgG2c, and **(C)** IgG1. **(D)** Antigen-specific long-lived antibody-secreting cells (ASCs) in bone marrow. Bars show mean and SD. Data were log-transformed and analyzed by one-way ANOVA with Sidak’s or Tukey’s correction for multiple comparisons.

In addition to cellular immune responses, we evaluated antibody-based responses in the immunized mice. Antigen-specific IgG, IgG2c, and IgG1 levels for all QS-21 liposome formulations tested were significantly higher than negative controls (**Figures 6A–C**). Similar to the cellular immune response patterns described above, there was a consistent trend for the addition of the TLR4 agonist GLA to increase IgG and IgG2c antibody responses compared to the QS-21 liposomes alone, but this increase in response was only significant in the case of canonical QS-21 liposomes since responses induced by the QS-21-Rha liposomes alone were already slightly elevated. The magnitude of the IgG2c and IgG1 antibody response induced by the canonical QS-21 and QS-21-Rha formulations indicated a balanced antibody response (**Figures 6B, C**, roughly 1:1) whereas the response was skewed towards a Th1-type response (higher IgG2c and lower IgG1) upon addition of GLA to either group, consistent with previous experience evaluating similar adjuvant combinations (23).

Recipients of either of the QS-21 liposome formulations revealed significant increases in the number of antibody-secreting plasma B cells in the bone marrow relative to recipients of the blank liposomes (although not relative to the saline control), and the addition of GLA to the formulations further augmented this response (**Figure 6D**). The number of antibody-secreting B cells found in the bone marrow was significantly higher in recipients of the QS-21-Rha liposomes compared to the canonical QS-21 liposomes, though the addition of GLA to the formulations led to equivalent responses in both and significantly higher than both negative controls. Together, the antibody and B cell data indicate that the most potent and durable humoral responses are induced by formulations containing GLA and either QS-21-Rha or canonical QS-21.

Overall, the liposomes containing QS-21-Rha elicited a well-balanced immune response although with a stronger Th1 quality compared to canonical QS-21 liposomes. Addition of the TLR4 agonist GLA generally increased response magnitudes and skewed responses further towards Th1, but in the case of liposomes containing QS-21-Rha, the added benefit of GLA was not always statistically significant due to the already elevated response induced by QS-21-Rha liposomes alone. In other words, the immunogenicity results generated by the formulations containing QS-21-Rha appeared to have distinctive qualities compared to the formulations containing canonical QS-21. However, additional studies are recommended to confirm these findings and benchmark to additional controls.

### Industrial impact of leaf-derived QS-21-Rha

The findings of this study indicate that QS-21-Rha represents a significantly more abundant and cost-effective source of vaccine adjuvant compared to the traditional canonical QS-21, which is obtained exclusively from the bark of aging, ecologically vulnerable *Q. saponaria* trees in Chile. Young *Q. saponaria* shrubs cultivated in California can produce at least 1.5 kg of dry aerial biomass annually. With conservative assumptions—6% w/w total saponins in the dry biomass and at least 10% w/w QS-21-Rha within that saponin fraction—a 2–3-year-old shrub yields approximately 9 g of QS-21-Rha/year. At a 60% recovery rate during purification, this translates to ~5.4 g of highly purified material per plant. By comparison, bark yields are substantially lower. On a dry weight basis, bark contains roughly 5% total saponins, of which about 60% can be recovered during purification to give ~3% purified saponins. Within this purified fraction (e.g., Vet-Sap, Desert King International), the final yield of canonical QS-21 is approximately 2% w/w (6), corresponding to an overall yield of only ~0.06% w/w canonical QS-21 from the starting bark material.

Scaling to production quantities, 1 kg of QS-21-Rha per year could be obtained from the aerial biomass of roughly 200 shrubs, whereas achieving the same from bark would require ~1700 kg of bark—entailing the felling and debarking of 100–120 mature wild trees (>30 years old). Crucially, aerial leaves and twigs can be harvested repeatedly without killing the shrubs, enabling a truly renewable agricultural model over extractive harvesting. An additional advantage is the chemical definition of QS-21-Rha, which can be isolated as a single, well-characterized component. Bark-derived extracts instead contain complex mixtures of QS-21 isomers and related compounds, including process-related impurities such as the m/z 2018 species (7), which can persist even after advanced purification. A single defined component greatly simplifies downstream processing and improves quality assurance.

From a production standpoint, QS-21-Rha derived from the leaves and twigs of *Q. saponaria* offers clear and immediate advantages over alternative manufacturing platforms such as plant cell culture or synthetic biology. Current engineered yeast or transient plant systems achieve QS-21 titers of only ~0.9–21 mg L^−1^ (5, 10), with similar yields reported for plant cell culture (~0.9 mg L^−1^). (11) These biotechnological approaches remain constrained by inherently low productivity, high media and energy costs, and substantial capital investment requirements, all of which translate into high unit costs and limited production volumes. At such titers, even a 10,000 L commercial fermenter would produce only ~100 g of canonical QS-21 before accounting for purification losses—orders of magnitude less than the multi-kilogram annual yields achievable from repeated, sustainable harvesting of field-grown aerial biomass. This positions leaf-based QS-21-Rha as a more sustainable, economically accessible, and rapidly scalable option for vaccine adjuvant manufacture. Taken together, this agricultural route alleviates pressure on wild *Q. saponaria* populations in Chile, delivers superior sustainability and cost-efficiency, and enhances product purity—factors likely to broaden the future use of QS-21-based vaccine adjuvants.

## Conclusion

This work represents the first successful isolation, purification, and immunological validation of QS-21-Rhamnose (QS-21-Rha) as a vaccine adjuvant. Although QS-21-Rha has been previously recognized as a minor (5–10%) component in bark extracts and known to occur in higher proportions in aerial biomass, no prior work had purified it to homogeneity or assessed its immunological activity independently of the canonical QS-21 mixture. Our findings demonstrate that QS-21-Rha can be efficiently extracted from the leaves and twigs of young plants grown in California, achieving >98% purity through chromatographic methods, thus eliminating the dependence on old-growth trees and establishing a renewable production platform.

The immunological evaluation revealed that QS-21-Rha formulated in liposomes elicits robust adaptive immune responses comparable to or exceeding those induced by canonical QS-21 variants. Specifically, QS-21-Rha demonstrated superior induction of CD4^+^ T cell activation markers, including significantly higher percentages of IFNγ^+^ CD4^+^ T cells and enhanced CD154^+^ (CD40L) expression, indicating strong T helper cell activation essential for effective antibody responses. ELISpot analyses also suggested greater cytokine-producing cell numbers, including some IL-17 and IL-5 response, suggesting distinct immunostimulatory qualities. In particular, as Th1 and Th17 responses are considered important to protect against tuberculosis, the QS-21-Rha adjuvant component could offer immunological as well as sustainability benefits for tuberculosis vaccine candidates (24, 25).

The implications for global vaccine manufacturing are substantial. Producing 1 kg of QS-21-Rha requires biomass from only ~200 shrubs—sustainably harvested without harming the plants—versus debarking ~100–120 wild *Q. saponaria* trees over 30 years old, a practice that is both ecologically damaging and supply-limited to Chile. This shift from extractive harvesting of endangered, slow-growing trees to renewable agricultural cultivation represents a paradigm change in the QS-21 supply chain.

Beyond the environmental benefits, the ability to obtain a single, chemically defined QS-21-Rha fraction—free from inseparable impurities such as the m/z 2018 species present in bark extracts—simplifies downstream processing, improves quality control, and has the potential to reduce manufacturing costs dramatically. By combining sustainability, scalability, chemical homogeneity, and proven immunological activity, QS-21-Rha from cultivated leaves provides a viable next-generation adjuvant component source. Adoption of this approach could secure long-term QS-21 supply, lower production costs, and support expanded global vaccine access, particularly in the context of pandemic preparedness and emerging infectious disease responses.

## Data Availability

The original contributions presented in the study are included in the article/**Supplementary Material**. Further inquiries can be directed to the corresponding authors.
