# Supplementary material for: Structure and immunological activity of QS-21 variant from Quillaja saponaria aerial biomass
Source: Front Immunol. 2026 Mar 9;17:1771912. doi: 10.3389/fimmu.2026.1771912 (PMC13006313; doi:10.3389/fimmu.2026.1771912)
Supplement: Supplementary file 1 [file DataSheet1.docx]

**Immunologically Active QS-21 Variant from *Quillaja saponaria* Aerial Biomass**

Ricardo San Martín^1,^*, Yuzhong Liu^2,^*, Yihan Yang^2^, Christopher B. Fox^3,5^, L. Ravi Iyer^3^, Raodoh Mohamath^3^, Gabi Ramer-Denisoff^3^, Robert Kinsey^3^, Jeffrey A. Guderian^3^, Jessica Schwabach^1^, Siwen Deng^1^, Natalia de Andrade Teixeira Fernandes^1^, Clayton Radke^4^

**Affiliations**¹ Sutardja Center for Entrepreneurship and Technology, College of Engineering, University of California, Berkeley, CA, USA
² Department of Chemistry, The Scripps Research Institute , La Jolla, CA, USA
³ Access to Advanced Health Institute (AAHI), Seattle, WA, US

^4^ Department of Chemical and Biomolecular Engineering, College of Chemistry, University of California, Berkeley, CA, USA

^5^ Department of Biological Engineering, College of Engineering, Utah State University, Logan, UT, USA

*These authors contributed equally to this work.

**Table of Content**

**Supplementary Table 1. List of *Q. saponaria* saponins in mature tree bark………S3**

**Supplementary Table 2. List of MS2 fragment ion masses and their corresponding intensities…………………………………………………………………………….….…….S4Supplementary Table 3. Percentage of QS-21-Rha in leaves………………….……..S6**

**Supplementary Table 4. Vaccine preparation for *in vivo tests*…………………………………….……..S7**

**Supplementary Table 5. Experimental timeline and readouts for mouse immunogenicity assay ………………….…….S8**

**Supplementary Figure 1. ^1^H NMR spectra of QS-21 and QS-21-Rha…………….….S9**

**Supplementary Figure 2. ^1^H NMR spectrum of QS-21-Rha…………………….…….S10**

**Supplementary Figure 3. ^13^C NMR spectrum of QS-21-Rha…………………….…..S11**

**Supplementary Figure 4. HSQC-TOCSY spectrum of QS-21-Rha………………….S12**

**Supplementary Figure 5. HSQC spectrum of QS-21-Rha……………………………S13**

**Supplementary Figure 6. HMBC spectrum of QS-21-Rha……………………………S14**

**Supplementary Figure 7. Liposome particle size.……………………………………..S15**

**Supplementary Figure 8. *In vitro* MCP production………………..………………….S16**

**Supplementary Figure 9. Adaptive memory CD4+ T cell responses with QS-21-Rha liposomes…………………………………………………………………………….….……S17**

**Supplementary Figure 10. Representative gating strategy.………………….………S18**

**Table S1.** *Q. saponaria* saponins identified in aqueous extracts of mature bark (> 30 y old) using UPLC/MS.

| **Retention Time (min)** | **Saponin** | **m/z** | **% of total saponins** |
| --- | --- | --- | --- |
| 3.812 | QS-7-Rha | 1876.2 | 1.35% |
| 3.883 | QS-7-Xyl | 1863.2 | 3.41% |
| 6.566 | QS-17-Rha | 1155.2 (half of 2310.0) | 4.16% |
| 6.749 | QS-17-Xyl | 1148.0 (half of 2296.0) | 7.71% |
| 7.914 | QS-18-Rha | 2165.6 | 7.30% |
| 8.171 | QS-18-Xyl | 2150.8 | 26.47% |
| 9.696 | QS-21-Rha | 2002.6 | 4.91% |
| 9.991 | QS-21-Xyl | 1988.4 | 12.51% |

**Table S2.** **MS2 fragment ion masses and their corresponding intensities**

| m/z | Abund | Abund % |
| --- | --- | --- |
| 469.1549 | 1225.76 | 36.6 |
| 469.1657 | 361.55 | 10.8 |
| 485.3231 | 534.24 | 15.95 |
| 485.3384 | 232.96 | 6.96 |
| 553.3473 | 377.48 | 11.27 |
| 553.3542 | 383.64 | 11.45 |
| 555.1917 | 216.02 | 6.45 |
| 563.3338 | 219.29 | 6.55 |
| 567.3278 | 543.24 | 16.22 |
| 567.3361 | 284.15 | 8.48 |
| 581.349 | 279.21 | 8.34 |
| 727.4028 | 244.73 | 7.31 |
| 761.411 | 307.4 | 9.18 |
| 789.3977 | 376.45 | 11.24 |
| 907.4611 | 465.89 | 13.91 |
| 907.481 | 182.1 | 5.44 |
| 951.4501 | 214.89 | 6.42 |
| 969.4661 | 3349.16 | 100 |
| 969.4884 | 681.27 | 20.34 |
| 970.4697 | 596 | 17.8 |
| 1525.6363 | 297.08 | 8.87 |
| 1525.6623 | 575.44 | 17.18 |
| 1567.6745 | 1566.38 | 46.77 |
| 1568.6799 | 776.95 | 23.2 |
| 1568.7059 | 171.61 | 5.12 |
| 1569.6842 | 238.35 | 7.12 |
| 1661.7465 | 504.74 | 15.07 |
| 1661.7682 | 421.88 | 12.6 |
| 1662.7557 | 315.32 | 9.41 |
| 1679.757 | 186.1 | 5.56 |
| 1739.7828 | 412.66 | 12.32 |
| 1983.9135 | 393.56 | 11.75 |
| 2001.8952 | 291.37 | 8.7 |
| 2001.9218 | 582.06 | 17.38 |
| 2001.9527 | 266.7 | 7.96 |
| 2002.9194 | 293.45 | 8.76 |
| 2002.9338 | 235.23 | 7.02 |
| 2003.9269 | 233.36 | 6.97 |
| 2948.9339 | 227.57 | 6.79 |

**Table S3.** Percentage of QS-21-Rha (% w/w dry biomass) in leaves of shrubs growing at various indoor and outdoor locations, leaves and bark of older wild trees.

|  |  |  |  | **QS-21-Rha** | |
| --- | --- | --- | --- | --- | --- |
| **Location/Type** | **Growth condition** | **Sample size** | **Total saponin % w/w dry biomass** | **Average QS-21-Rha % of total saponins^1^** | **Average content QS-21 % w/w dry biomass** |
| **Leaves, Berkeley, California** | Field- 2.5 y old | 29 | 10.1% | 23.2% | 2.3% |
| **Leaves, Berkeley, California** | Lath house- 2.5 y old | 14 | 7.0% | 20.6% | 1.4% |
| **Leaves, Berkeley, California** | Greenhouse 2.5 y old | 49 | 11.2% | 12.9% | 1.5% |
| **Leaves, Berkeley, California** | Wild trees, > 50 y old | 6 | 4.0% | 14% | 0.6% |
| **Leaves, Santa Barbara, California** | Wild trees, > 30 y old | 13 | 2.2% | 16% | 0.3% |

**Notes:** Total saponin w/w percentage based on dry biomass. % QS-21-Rha: percentage of total saponins detected by UPLC-MS.

**Table S4.** Vaccine preparation for *in vivo* tests.

| **Group #** | **Mice #** | **Description** | **ID93 dose (µg)** | **Canonical QS-21 dose**  **(µg)** | **QS-21-Rha dose**  **(µg)** | **GLA dose**  **(µg)** |
| --- | --- | --- | --- | --- | --- | --- |
| 1 | 4 | Saline (Negative control) | 0.5 | - | - | - |
| 2 | 8 | ID93 + canonical QS-21 liposomes | 0.5 | 2 | - | - |
| 3 | 8 | ID93 + QS-21-Rha liposomes | 0.5 | - | 2 | - |
| 4 | 8 | ID93 + canonical GLA-QS-21 liposomes | 0.5 | 2 | - | 5 |
| 5 | 8 | ID93 + GLA-QS-21-Rha liposomes | 0.5 | - | 2 | 5 |
| 6 | 8 | ID93 + blank liposomes | 0.5 | - | - | - |

**Table S5.** Experimental timeline and readouts for mouse immunogenicity assay

| **Activity** | **Day** | **notes** |
| --- | --- | --- |
| Prime immunization (intramuscular) | 0 | Bilateral in 100 µL total volume, administered into hind quadriceps. Blood sample taken. |
| Booster immunization (intramuscular) | 21 | Bilateral in 100 µL total volume, administered into hind quadriceps. Blood sample collected prior to immunization. |
| Study termination and tissue harvest | 35 | Blood collected for IgG, IgG2c, IgG1 antibody titer analysis. Spleen collected for intracellular cytokine staining flow cytometry and ELISpot analysis. Bone marrow collected for long-lived plasma cell ELISpot analysis. |


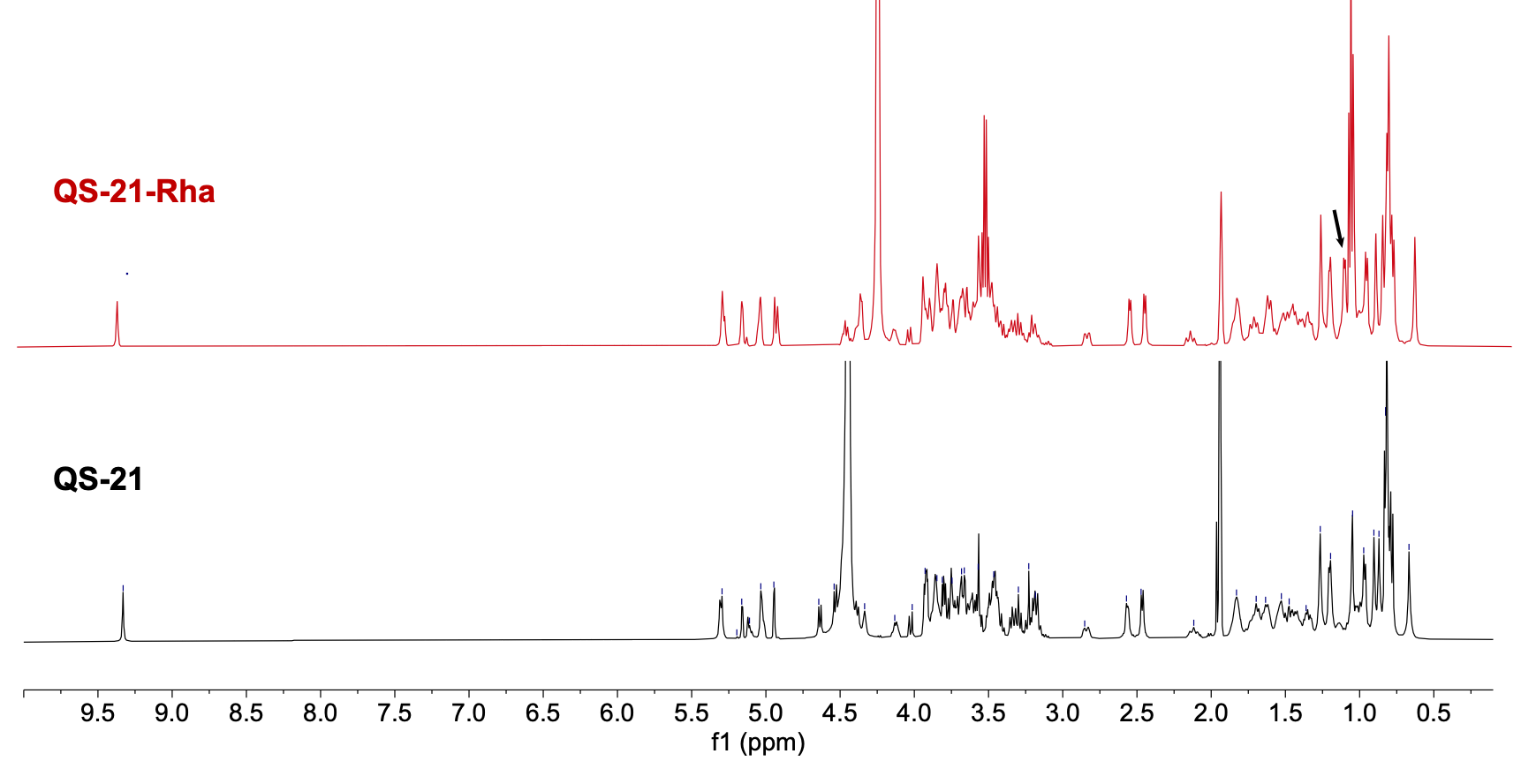


**Figure S1.** ^1^H NMR spectra of QS-21 and QS-21-Rha. QS-21-Rha displays an additional methyl doublet resonance at ~1.1 ppm, diagnostic of the C6 methyl group of rhamnose, absent in the QS-21 spectrum.


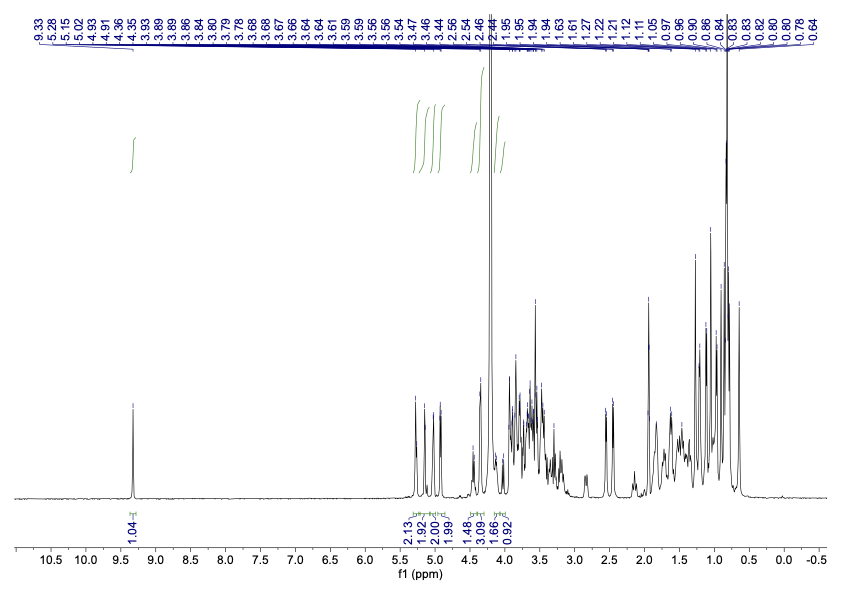


**Figure S2.** ^1^H NMR spectra of QS-21-Rha.


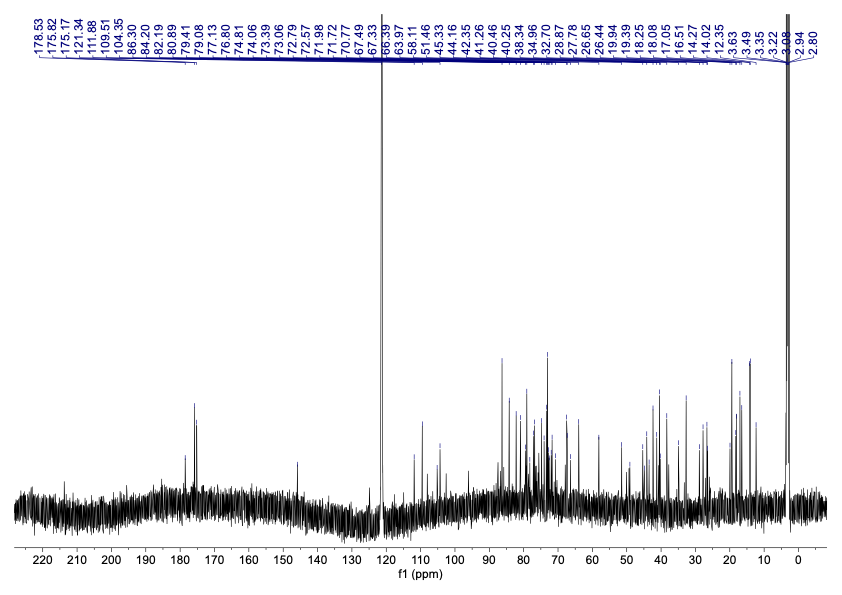


**Figure S3.** ^13^C NMR spectra of QS-21-Rha.


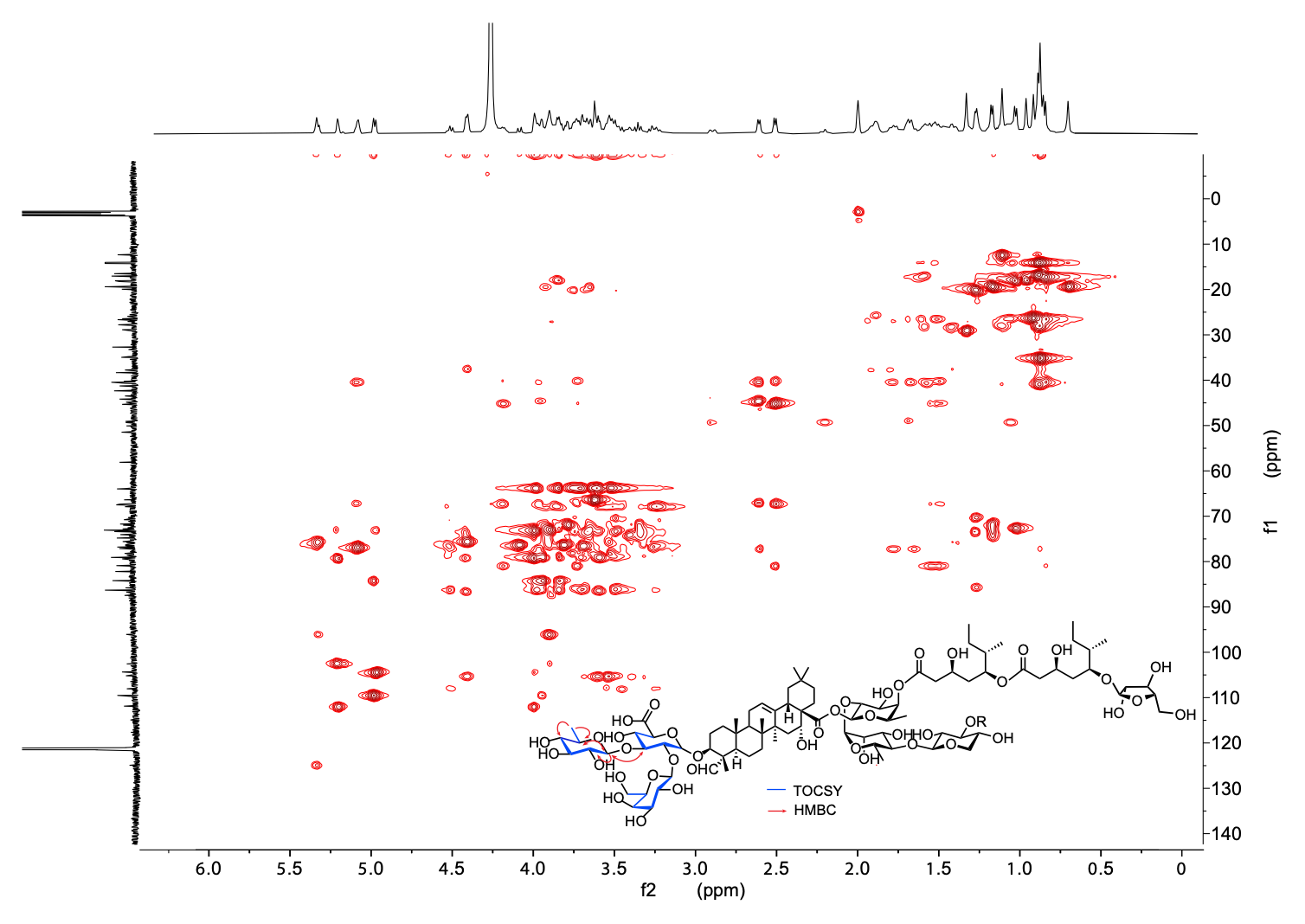


**Figure S4.** HSQC-TOCSY correlations used to assign the rhamnose spin system in QS-21-Rha.


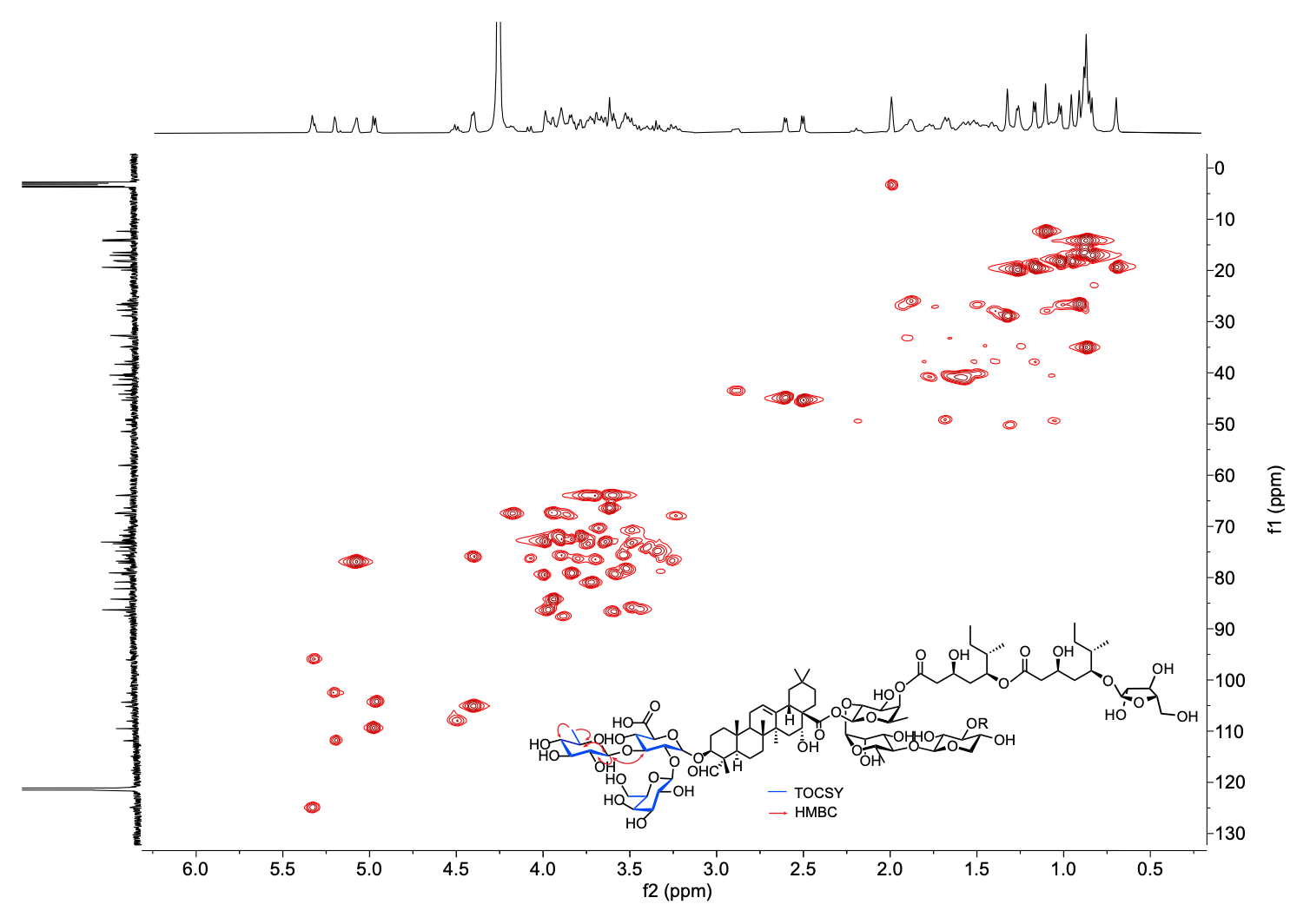


**Figure S5.** HSQC spectra of QS-21-Rha.


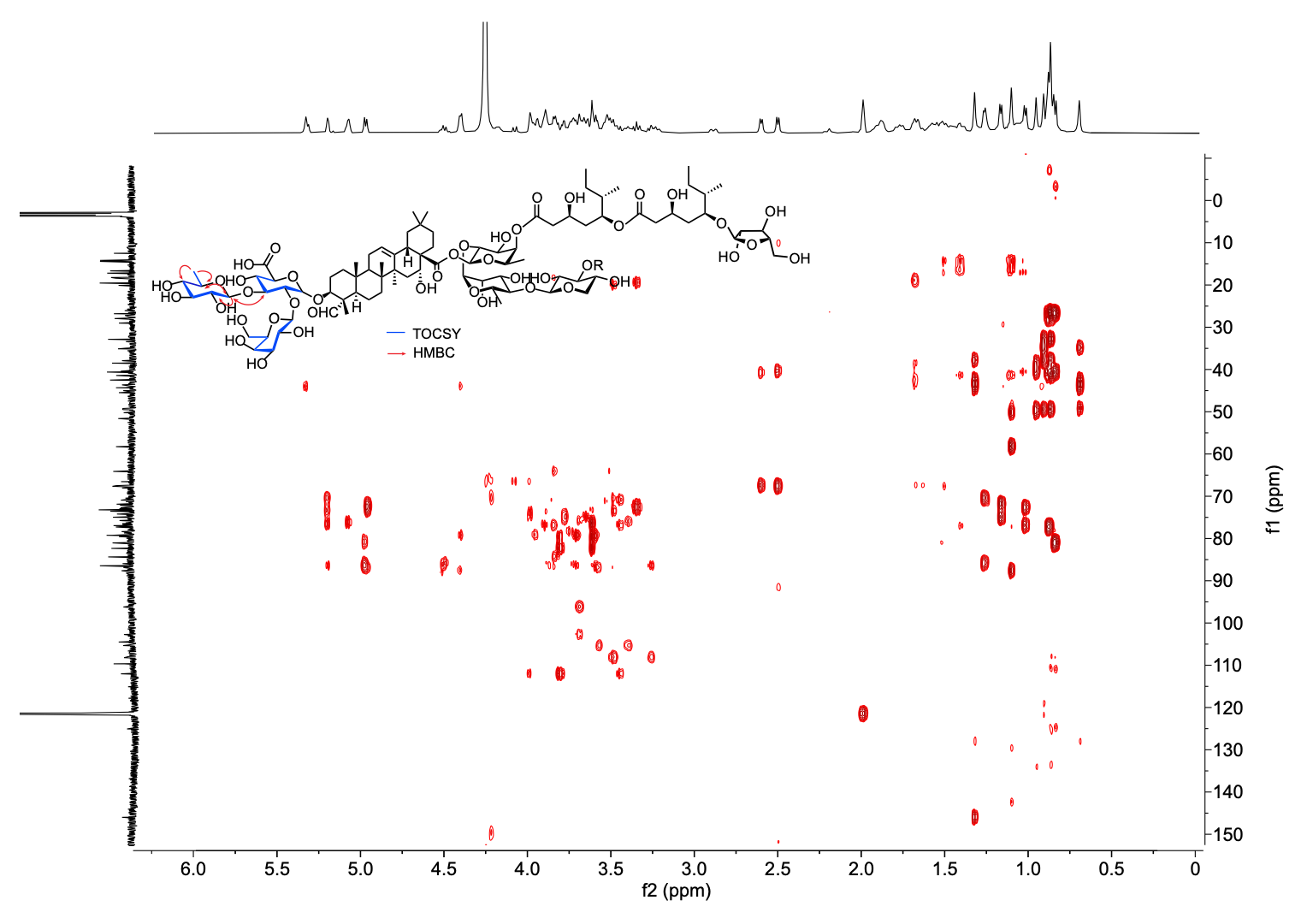


**Figure S6.** HSQC spectra of QS-21-Rha.


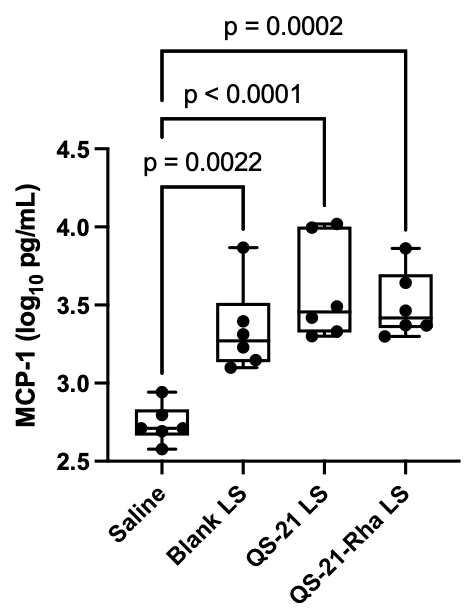


**Figure S7.** *In vitro* MCP-1 production from liposomes containing 4 µg/mL of QS-21 liposomes vs QS-21-Rha liposomes (LS). Values represent human whole blood samples from 6 donors (3 male and 3 female). Data are represented as box-whisker plots with bars representing median values, boxes representing 1st–3rd quartiles, and whiskers representing the maximum and minimum values. Data were log-transformed and analyzed by one-way ANOVA with Tukey’s correction for multiple comparisons.


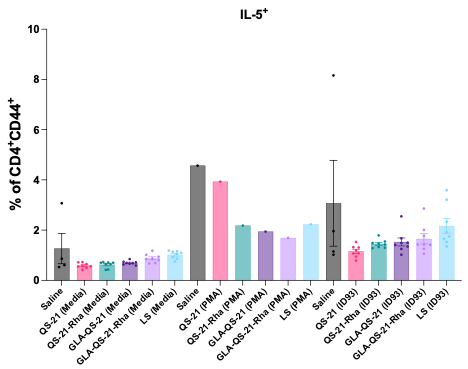


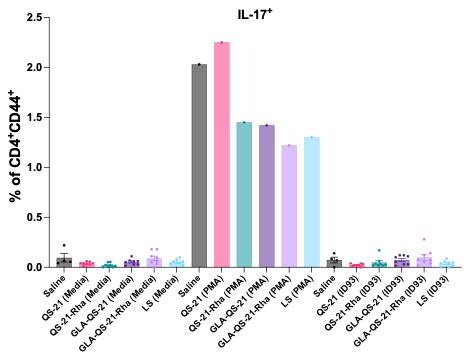


**Figure S8.** Adaptive ID93-specific CD4+ T cell responses in mice immunized with ID93 + QS-21-Rha liposomes (UC Berkeley) vs QS-21 liposomes (AAHI). C57BL/6 mice (*n* = 8/group except for saline control with *n* = 4/group) were immunized on days 0 and 21, and spleens were harvested on day 35 for intracellular cytokine staining analysis. Medium was used for negative controls, and PMA-ionomycin was used for positive controls. Means and SEM are shown. Statistical analysis by ordinary one-way ANOVA with Tukey’s test for multiple comparisons.


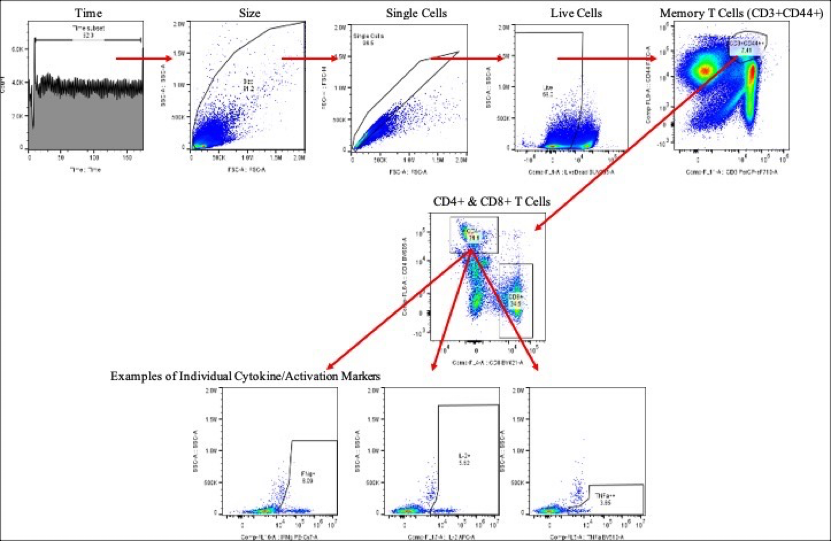


**Figure S9.** Representative gating strategy.
